# Liver gene therapy with intein‐mediated F8 *trans*‐splicing corrects mouse haemophilia A

**DOI:** 10.15252/emmm.202115199

**Published:** 2022-05-02

**Authors:** Federica Esposito, Hristiana Lyubenova, Patrizia Tornabene, Stefano Auricchio, Antonella Iuliano, Edoardo Nusco, Simone Merlin, Cristina Olgasi, Giorgia Manni, Marco Gargaro, Francesca Fallarino, Antonia Follenzi, Alberto Auricchio

**Affiliations:** ^1^ Telethon Institute of Genetics and Medicine (TIGEM) Pozzuoli Italy; ^2^ Department of Health Sciences University of Piemonte Orientale “Amedeo Avogadro” Novara Italy; ^3^ Department of Medicine and Surgery University of Perugia Perugia Italy; ^4^ Medical Genetics Department of Advanced Biomedical Sciences Federico II University Naples Italy

**Keywords:** AAV vectors, haemophilia A, liver gene therapy, protein *trans*‐splicing, Genetics, Gene Therapy & Genetic Disease, Haematology, Methods & Resources

## Abstract

Liver gene therapy with adeno‐associated viral (AAV) vectors is under clinical investigation for haemophilia A (HemA), the most common inherited X‐linked bleeding disorder. Major limitations are the large size of the *F8* transgene, which makes packaging in a single AAV vector a challenge, as well as the development of circulating anti‐F8 antibodies which neutralise F8 activity. Taking advantage of split‐intein‐mediated protein *trans*‐splicing, we divided the coding sequence of the large and highly secreted F8‐N6 variant in two separate AAV‐intein vectors whose co‐administration to HemA mice results in the expression of therapeutic levels of F8 over time. This occurred without eliciting circulating anti‐F8 antibodies unlike animals treated with the single oversized AAV‐*F8* vector under clinical development. Therefore, liver gene therapy with AAV‐*F8*‐N6 intein should be considered as a potential therapeutic strategy for HemA.

## Graphical abstract

Liver directed AAV‐intein mediated protein trans‐splicing results in stable therapeutic levels of F8 in mice thus representing a novel therapeutic strategy for haemophilia A.

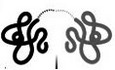

**The paper explained**

**Medical issue**

With an incidence of 1 in 5,000 males, haemophilia A (HemA) is the most common inherited bleeding disorder which is caused by the deficiency of coagulation factor VIII (F8). The size of F8 coding sequence exceeds the cargo capacity of adeno‐associated viral (AAV) vectors; therefore, current HemA gene therapy clinical trials use AAV with oversize genomes.

**Results**

We tested if intein‐mediated protein trans‐splicing (PTS) allows to reconstitute F8 in mouse liver transduced by AAV, thus overcoming the limitations imposed by the vector transfer capacity. We show that AAV‐intein reconstitute F8 to therapeutic levels in HemA mice and this occurs without the development of anti‐F8 antibodies at the vector doses tested.

**Clinical impact**

Liver gene therapy with AAV‐intein represents a potential therapeutic strategy for HemA using relatively low doses of vectors with defined genomes which do not elicit anti‐F8 antibodies.

## Introduction

Haemophilia A (HemA) is the most common inherited X‐linked recessive coagulation disorder caused by the partial or complete deficiency of coagulation F8. F8 activity levels are inversely proportional to bleeding risk; severely affected patients (about 50% of all cases) have circulating protein levels of < 1% (Antonarakis *et al*, 1995; White *et al*, 2001; Bowen, 2002). Levels of F8 activity between 1 and 5% result in a moderate phenotype, levels between 5 and 50% give a mild phenotype, and levels above 50% are associated with normal haemostasis (White *et al*, 2001).

The current management of HemA involves prophylactic administration of recombinant or plasma‐derived F8. Lifelong intravenous infusions are required as often as 2–3 times weekly in severely affected patients. The chances of developing neutralising anti‐F8 antibodies (inhibitors) remain high with about 30% of patients having to discontinue treatment (Cafuir & Kempton, 2017). This is potentially overcome by the recently approved bispecific antibody Emicizumab, which has significantly broadened the treatment options for HemA, allowing treatment of even younger patients which was previously unfeasible with standard care (Mahlangu *et al*, 2018; Oldenburg *et al*, 2018). Emicizumab is injected subcutaneously every 1–4 weeks with almost no bleeding occurring in the majority of patients. However, the management of spontaneous bleeds on Emicizumab still requires standard infusions (Butterfield *et al*, 2019).

Regardless of the various existing treatment options, the possibility of curing HemA rather than managing the disease remains the hope of many patients. In the last two decades, gene therapy for HemA has been under extensive investigation after it was observed that even modest improvements in the F8 levels (by 1–2%) can significantly reduce the risk of spontaneous bleeding events and the need for F8 replacement infusions (Manco‐Johnson *et al*, 2007).

Adeno‐associated viral (AAV) vectors have emerged as the most promising *in vivo* gene therapy approach for HemA, because of their excellent safety profile and their ability to direct long‐term transgene expression from post‐mitotic tissues such as the liver (Nathwani *et al*, 2004, 2017). However, HemA poses a great challenge to AAV gene therapy because of the size (7 kb) of the *F8* coding sequence (CDS) to be transferred which exceeds the canonical AAV cargo capacity of ~4.7 kb. For this reason, all of the AAV‐based products under clinical investigation consist of B‐domain‐deleted (BDD) versions of the *F8* transgene, which are ~4.4 kb in size (Makris, 2020). However, using a transgene of this size leaves limited space in the vector for the necessary regulatory elements, thus restricting the choice of promoters and polyA signals. Moreover, all these vector genomes are on the verge of AAV’s normal cargo capacity and are thus at risk of being improperly packaged as a library of heterogeneous truncated genomes. Despite the ability of such oversize vectors to successfully express large proteins, their long‐term efficiency and safety are yet to be confirmed (Grieger & Samulski, 2005; Dong *et al*, 2010; Hirsch *et al*, 2010; Wu *et al*, 2010).

As an alternative, different groups have explored strategies based on co‐delivery of dual AAV vectors to reconstitute *F8*. Each vector encodes for one of the two chains, which should then re‐associate and produce the biologically active heterodimer F8. However, the main drawback of this approach is the apparent chain imbalance, which derives from less efficient secretion of the heavy chain than the light one. This results in the production of higher amounts of inactive protein compared with full‐length F8 (Burton *et al*, 1999; Scallan *et al*, 2003; Chen *et al*, 2009; Zhu *et al*, 2012).

More recently, protein *trans*‐splicing (PTS) has been evaluated to reconstitute large proteins via AAV vectors. PTS is used by unicellular organisms across all three domains of life to reconstitute large proteins from shorter precursors that include split‐inteins (intervening proteins) at their extremities. Following translation, split‐inteins mediate their association and self‐excision from the host protein in an independent process that does not require any energy supply (Mills *et al*, 2014; Li, 2015). PTS has been used with limited success to reconstitute F8 (Chen *et al*, 2007), while we have recently shown that AAV‐intein‐mediated PTS in the retina results in therapeutic levels of protein reconstitution which in some instances match those achieved by single AAV vectors (Tornabene *et al*, 2019).

Encouraged by these results, we explored the efficiency of liver gene therapy with AAV‐intein vectors to reconstitute the large and highly active F8‐N6 variant (N6) (5 kb) (Miao *et al*, 2004; Ward *et al*, 2011).

## Results

### AAV‐intein‐mediated protein *trans*‐splicing in mouse liver

**Figure 1:**
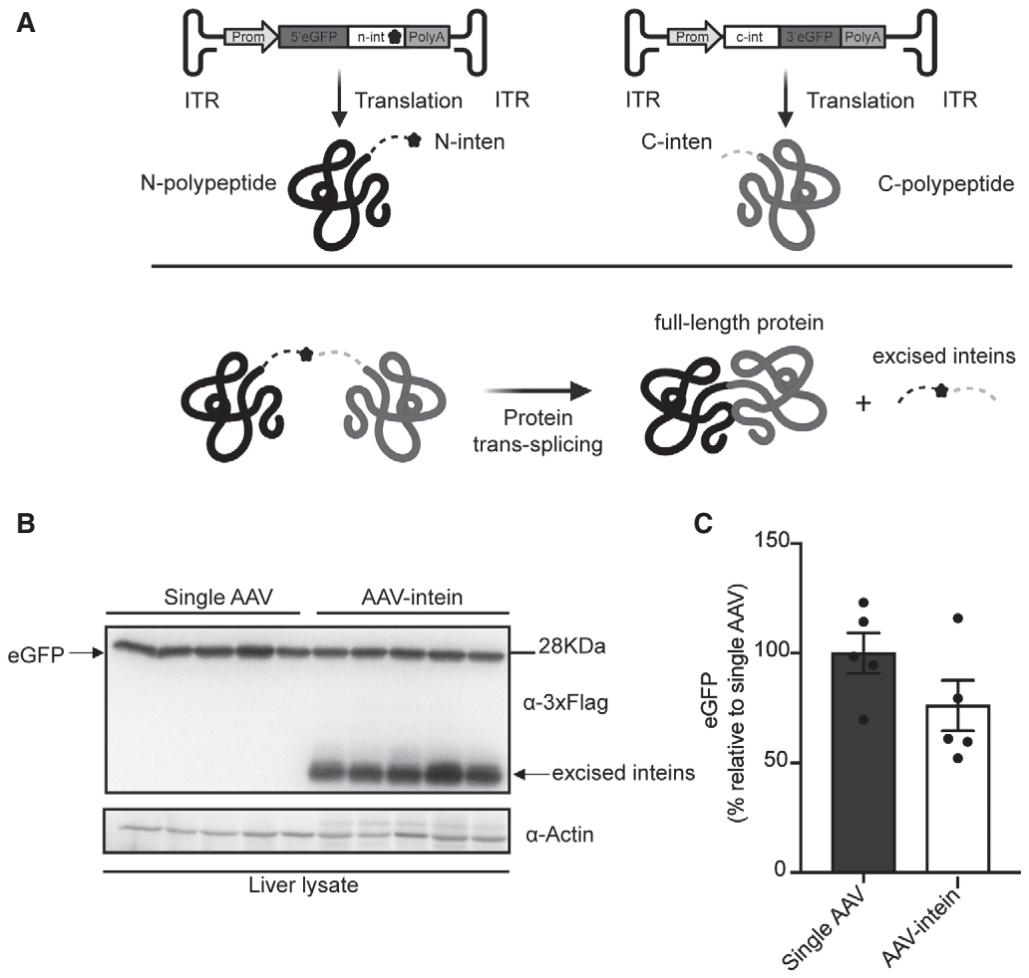
Intein‐mediated protein *trans*‐splicing in liver Schematic representation of the enhanced green fluorescent protein (eGFP) intein constructs and of the intein‐mediated protein *trans*‐splicing. ITR—inverted terminal repeats; Prom—promoter; 5’eGFP—5’eGFP coding sequence (CDS); n‐intein—N‐terminal of DnaE intein; star symbol—3xFlag tag; PolyA—short synthetic polyadenylation signal; c‐intein—C‐terminal of DnaE intein; 3’eGFP—3’ eGFP CDS.Western blot analysis of liver lysates (100 µg) shows that intein‐mediated protein *trans*‐splicing efficiently reconstitutes full‐length eGFP. Single AAV: *n* = 5; AAV‐intein: *n* = 5. Arrows indicate both full‐length eGFP and excised inteins.Quantification of eGFP protein bands. Values are reported as mean ± SEM. Each dot represents the eGFP protein band quantification from animals injected with either single AAV *n* = 5 or AAV‐intein *n* = 5. Source data are available online for this figure.

We assessed the potential of split‐intein‐mediated protein *trans*‐splicing in liver by comparing the efficiency of adeno‐associated viral (AAV) vector intein to that of a single AAV vector. To do so, we used the reporter enhanced green fluorescent protein (eGFP) whose coding sequence (CDS) fits well within a single AAV. The full‐length eGFP CDS was divided into two separate halves at Cysteine 71 and fused to either the N‐ and C‐terminal halves of the DnaE split‐inteins from *Nostoc punctiforme* (*Npu*; Iwai *et al*, 2006) (Fig 1A). These were cloned under the human thyroxin binding globulin (TBG) liver promoter (Yan *et al*, 2012) and separately packaged into AAV8 vectors that efficiently target liver. A single AAV8 vector carrying the full‐length eGFP CDS under the same TBG promoter was produced for the *in vivo* comparison. Five‐week‐old C57/BL6 mice were injected retro‐orbitally with either the single or the two AAV‐intein vectors at the dose of 5 × 10^11^ GC of each vector per animal. Livers were harvested 4‐week post‐injection (4 wpi) and Western blot (WB) analysis of liver lysates followed by quantification of the eGFP bands showed that AAV‐intein reconstitute about 76% of the full‐length eGFP protein produced by the single vector (Fig 1B and C).

### *In vitro* characterisation of human F8 variants

**Figure 2:**
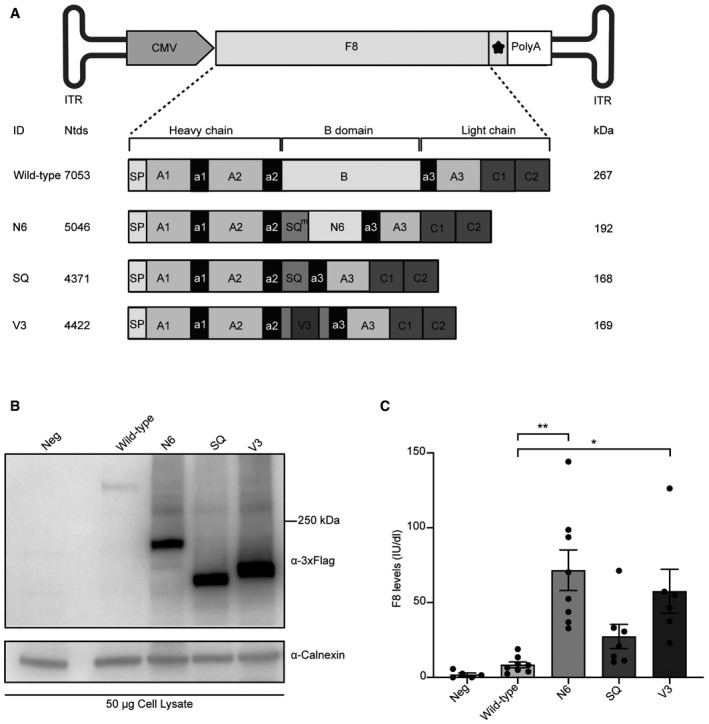
Comparison of human F8 variants *in vitro* Schematic representation of the four different F8 variants that were cloned into an AAV plasmid: wild‐type F8; N6 containing 11 amino acids of the modified SQ amino acid linker (SQ^m^) followed by the human N6 B domain; SQ containing the SQ amino acid linker; V3 containing the V3 peptide in the middle of the SQ linker. ITR—inverted terminal repeats; CMV—cytomegalovirus promoter; star symbol—3xFlag tag; PolyA—short synthetic polyadenylation signal; Ntds—nucleotides; SP—signal peptide. Details on the exact amino acid differences in the B domain can be found in Fig EV1.Western blot analysis of lysates of HEK293 cells 72‐h post‐transfection with the various F8 variants. Neg—non‐transfected cells.Chromogenic assay of F8 activity in the medium of transfected cells is reported as International Units/decilitre (IU/dl). Data are presented as mean ± SEM. Each dot within the same group corresponds to a biological replicate: Neg *n* = 5; Wild‐type *n* = 8; N6 *n* = 8; SQ *n* = 7; V3 *n* = 6. Significant differences between groups were assessed using the Kruskal–Wallis test followed by the *post hoc* analysis: Nemenyi’s All‐Pairs Rank Comparison Test. The Kruskal–Wallis test *P* = 2.88e‐05. **indicates the significant difference between the N6 and the Wild‐type groups: *P *≤ 0.01. *indicates the significant difference between the V3 and the Wild‐type groups: *P *≤ 0.05. All *P*‐values are reported in Appendix Table S1. Source data are available online for this figure.

**Figure EV1:**
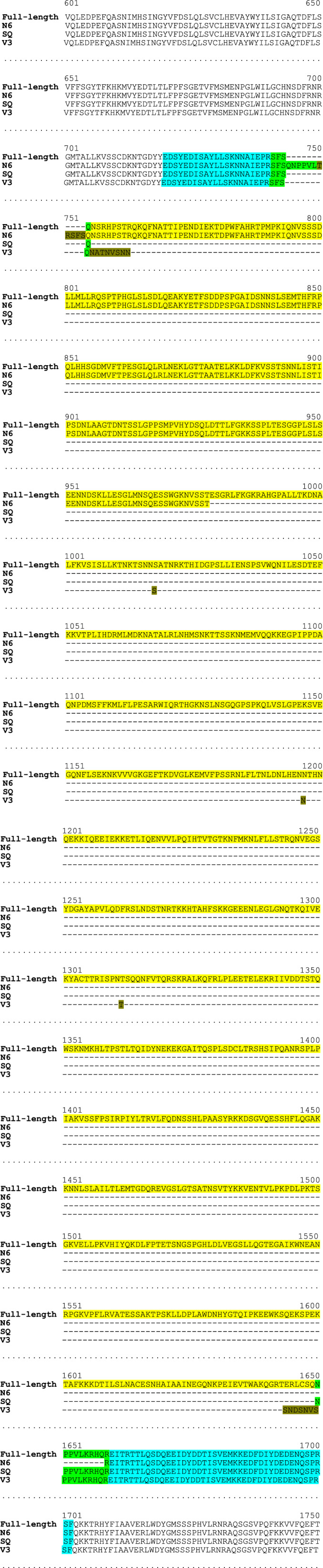
Amino acid sequence alignment underlining the differences in the B domain of the F8 variants The amino acid (aa) sequences of the B domain (or substitution linkers corresponding to it) of the F8 variants were aligned using the Clustal Omega software. a2 acidic linker of the F8 protein spans from aa position 720 to 740; the entire B domain or its substitutions are shown between aa 741 and 1659; a3 acidic linker spans from aa 1660 to 1702. Full‐length, wild‐type F8; N6, BDD F8‐N6; SQ, BDD F8‐SQ; V3, BDD F8‐V3.

After assessing that AAV‐intein transduce efficiently liver, we set up to select the best F8 transgenic variant to be expressed via AAV‐intein vectors. To this end, we compared the wild‐type F8 coding sequence (CDS) to 3 commonly used B‐domain‐deleted (BDD, which lack F8 amino acids from 740 to 1649 (Miao *et al*, 2004) versions (Figs 2A and EV1 for exact amino acid differences). Specifically, the 3 BDD constructs carry different codon‐optimised linkers in the place of the B domain, which are designed to promote efficient F8 secretion by mimicking some of the natural F8 post‐translational modifications (Miao *et al*, 2004): F8‐N6 (N6) contains 11 amino acids from the modified SQ activation peptide (SQ^m^) from Ward *et al* (2011), followed by the N6 human B domain spacer involving 6 (*N*)‐linked glycosylation sites (Miao *et al*, 2004); F8‐SQ (SQ) contains the original SQ linker described by Sandberg *et al* (2001). This variant is available for clinical use as a replacement recombinant F8 product (ReFacto, Wyeth Pharma; Toole *et al*, 1986) and is also under investigation in more than one AAV gene therapy clinical trial (Butterfield *et al*, 2019). The F8‐V3 variant (V3) consists of a small 17‐aa peptide, which contains the original 6 (*N*)‐linked glycosylation triplets from the N6 inserted into and flanked by the SQ linker (McIntosh *et al*, 2013). This variant has been previously described as another small version of BDD F8, able to achieve high levels of F8 activity in mice and non‐human primates (McIntosh *et al*, 2013) as well as in human subjects (Nathwani *et al*, 2018).

**Figure EV2:**
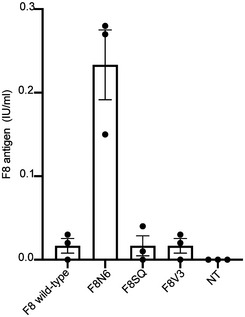
F8 antigen quantification in the media of transfected HEK293 cells Enzyme‐linked immunosorbent assay (ELISA) for F8 antigen performed on medium of HEK293 cells transfected with plasmid encoding for each hF8 variant (*n* = 3 biological replicates). F8 antigen levels are reported as International Units/millilitres (IU/ml). Data are represented as mean ± SEM. Source data are available online for this figure.

All four variants were independently cloned into an AAV backbone plasmid under the control of the CMV promoter, including both a short synthetic polyadenylation signal (Levitt *et al*, 1989) and a triple flag tag (3xFlag) to allow for easy detection of the proteins. The constructs were tested by transient transfection in the human embryonic kidney cell line 293 (HEK293). WB analysis of the cell lysates 72‐h post‐transfection (hpt) revealed bands of the expected size (Fig 2B). To detect the biological activity of each variant, cells were cultured for 12 h following transfection, after which they were kept in serum‐free medium until the time point of 72 hpt when F8 activity was measured by chromogenic assay on media of transfected cells. All variants produced detectable F8 activity levels (Fig 2C). Wild‐type F8 had fairly low mean levels of activity of 8.4 International Unit/decilitre (IU/dl), followed by SQ with 27.4 IU/dl, V3 with 57.6 IU/dl and N6 with the highest mean levels of 71.6 IU/dl. There was a significant difference in potency of the wild‐type F8 and both the N6 and V3 constructs, which was determined by the Kruskal–Wallis rank‐sum test; this difference was more significant for the N6 (**P ≤ 0.001) than for the V3 variant (*P ≤ 0.05). In addition, enzyme‐linked immunosorbent assay (ELISA) analysis of media from transfected cells showed that N6 is the most secreted F8 variant (Fig EV2). Based on this, we selected N6 as the variant to be tested by AAV‐intein in comparison with one of the traditional single AAV replacement gene therapy, which is under clinical investigation (*NCT03001830*).

### AAV‐intein‐mediated protein *trans*‐splicing efficiently reconstitutes N6 *in vitro*

**Figure 3:**
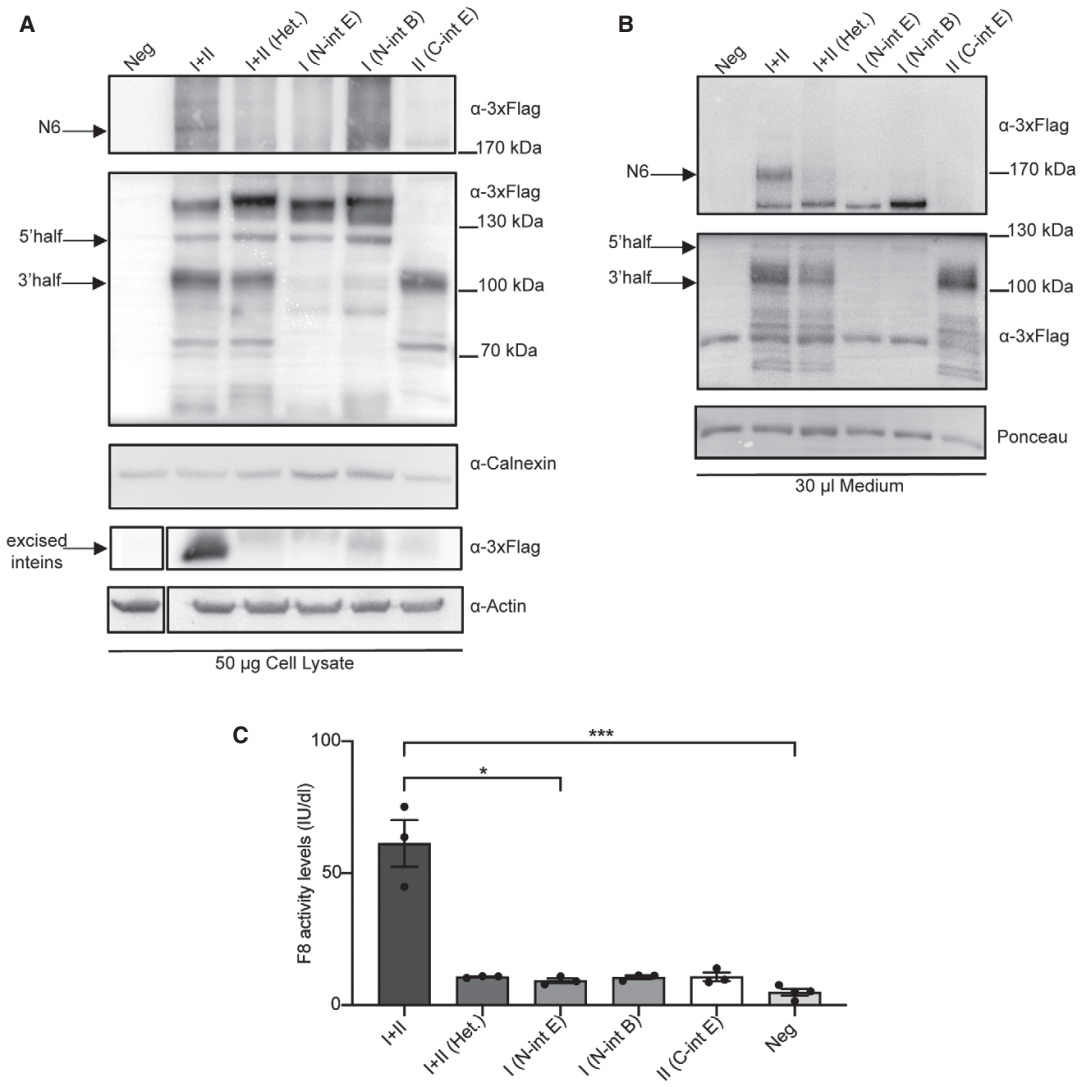
*In vitro* F8‐N6 (N6) intein expression and activity Western blot (WB) of protein lysates of HEK293 cells 72‐h post‐transfection (*n* = 3; biological replicates) with either *Npu* inteins or Heterologous (N‐intein DnaB + C‐intein DnaE) split‐inteins. I + II, N6 split‐intein proteins; I+II (Het.), heterologous split‐intein proteins; I, 5’N6 coding sequence (CDS)‐N‐DnaE protein; I (N‐int B), 5’N6 CDS‐N‐DnaB. II, C‐DnaE‐3’N6 CDS protein. Excised inteins (~12 kDa) are present only in the down part of the blot when I + II (*Npu* inteins) are provided. Arrows indicate the full‐length N6 protein, single halves and excised inteins.WB of medium of the transfected cells showing the secreted proteins (*n* = 3; biological replicates). Arrows indicate the full‐length N6 protein as well as single halves and excised inteins.Chromogenic assay performed on the medium of transfected cells to detect F8 activity levels reported as International Units/decilitre (IU/dl). Data are presented as mean ± SEM. Each dot within each group represents a different biological replicate (*n*): I + II *n* = 3; I + II (Het.) *n* = 3; I (N‐int E) *n* = 3; I (N‐int B) *n* = 3; II (C‐int E) *n* = 3; Neg *n* = 4. Significant differences between groups were assessed using Kruskal–Wallis rank‐sum test Kruskal−Wallis, *P* = 0.013. *indicates the significant difference between the I + II and the I (N‐int E) groups: *P *≤ 0.05. ***indicates the significant difference between the I + II and the Neg groups: *P *≤ 0.001. All *P*‐values are reported in Appendix Table S1. Source data are available online for this figure.

To test the efficiency of intein‐mediated N6 protein *trans*‐splicing (PTS), we split the large coding sequence (CDS) into two fragments, that is, the 5’ and 3’ half, fused, respectively, to the N‐ and C‐terminal halves of the DnaE split‐inteins from *Nostoc punctiforme* (*Npu*; Iwai *et al*, 2006). The split CDS was cloned into two separate AAV plasmids which included the same regulatory elements as above, together with a 3xFlag to detect both N6 halves as well as the full‐length protein and excised inteins. The splitting point was selected within the B domain, which is known to be dispensable for F8 expression and procoagulant activity (Pipe, 2009), thus aiming to preserve the integrity of the other more critical protein domains. To optimise the chosen splitting position, the intrinsic amino acid residue requirements for efficient protein *trans*‐splicing with the *Npu* inteins were also considered. Specifically, the main prerequisite is the presence of an amino acid containing either a thiol or hydroxyl group (Cysteine, Serine or Threonine) as the first residue in the 3’ half of the CDS (Shah *et al*, 2013; Cheriyan *et al*, 2014). The intein set was designed within the N6 linker (Ser962, considering the signal peptide) of the N6 variant. Moreover, to assess whether F8 activity in the medium of transfected cells was specifically due to the reconstitution of the full‐length N6 after PTS, a set of N6 flanked by heterologous split‐inteins was also designed. In this set, the N‐terminal of the 5’ half of N6 CDS was fused to the split N‐inteins DnaB from *Rhodothermus marinus* (*Rma*; Zhu *et al*, 2013; Tornabene *et al*, 2019) while the C‐terminal of the 3’half was fused to the split C‐intein DnaE. Both intein sets were tested by transient transfection into HEK293 cells. Seventy‐two‐hour post‐transfection, cell lysates and medium were harvested, and N6 expression was evaluated by WB (Fig 3A and B). Both full‐length N6 protein of the expected size (~190 kDa in cell lysate and ~170 kDa in the medium) and the excised DnaE inteins (~18 kDa) were detected only when the *Npu* inteins set was used. Quantification of the single halves after PTS shows that the 5’(~123 kDa) and 3’(~95 kDa) half are fivefold and fourfold more abundant than the full‐length N6, respectively.

The activity levels of the secreted F8 in the medium were found to be ~60 IU/dl on average, while single halves as well as the heterologous intein set exhibited little to no activity (Fig 3C).

### N6 codon optimisation increases F8 expression and activity *in vitro*

**Figure 4:**
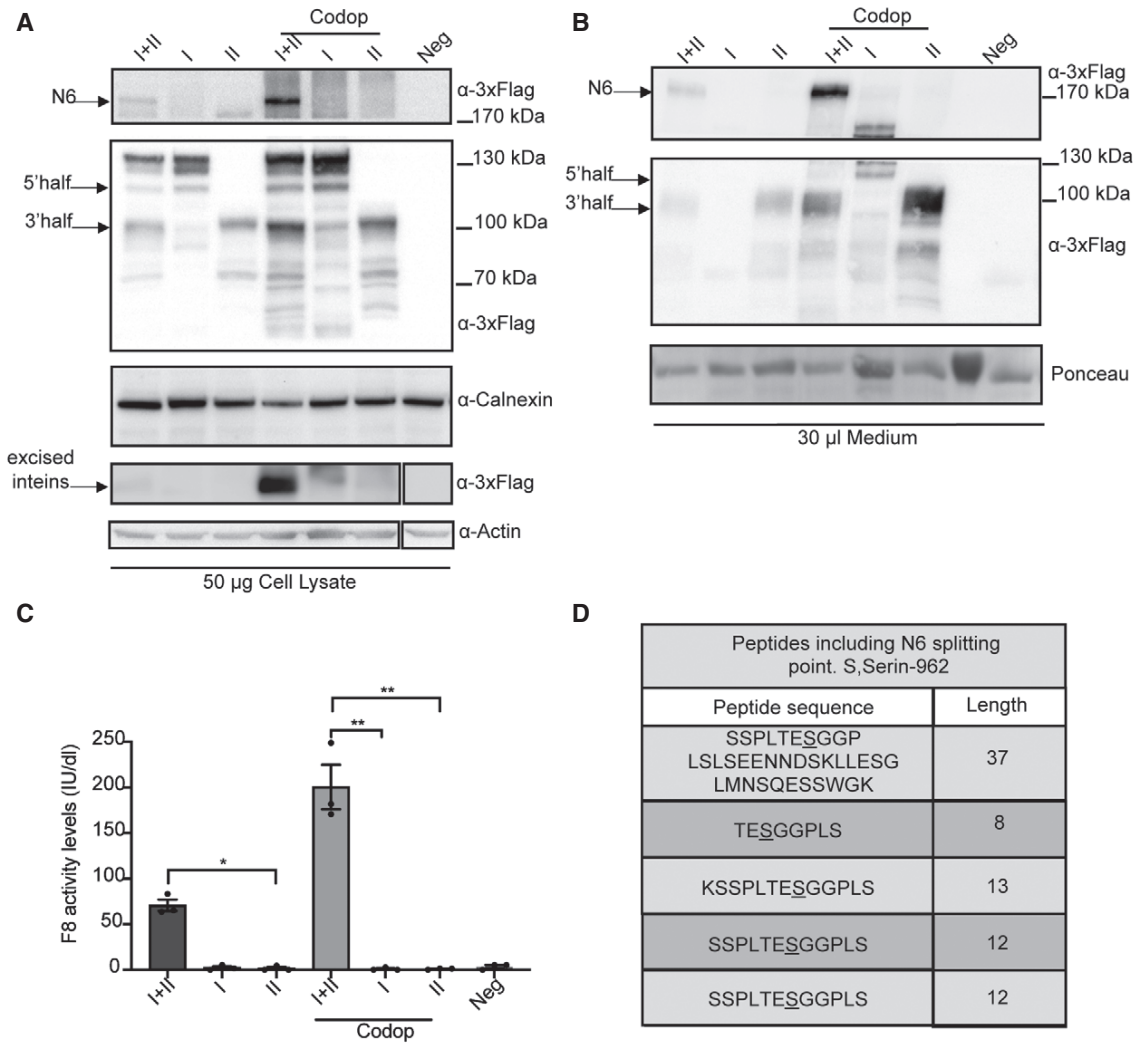
Codon optimisation of the N6 split‐intein improves F8 activity levels Western blot (WB) of protein lysates of HEK293 cells 72 hpt with the AAV‐N6 split‐intein plasmids and with the codon‐optimised set. I + II, N6 split‐intein proteins; I, 5’ N6 CDS‐N‐DnaE protein; II, C‐DnaE‐3’N6 CDS protein. (*n* = 3; biological replicates). Arrows indicate the full‐length N6 protein, excised inteins and both single halves. Codop: codon‐optimised.WB of medium from the transfected cells showing increased secretion of the CodopN6 full‐length protein compared with the non‐codon‐optimised. (*n* = 3; biological replicates). Arrows indicate the full‐length N6 protein, excised inteins and both single halves.Chromogenic assay performed on the medium from transfected cells to measure F8 activity levels reported as International Units/decilitre (IU/dl) (*n* = 3; biological replicates). Data are presented as mean ± SEM. Significant differences between groups were assessed using Kruskal–Wallis test *P* = 0.027. *indicates the significant difference between the I + II (N6 split‐intein) and the II (C‐DnaE‐3’N6 CDS) groups: *P *≤ 0.05. **indicates the significant difference between the Codop I + II and the I (5’ N6 CDS‐N‐DnaE) codop groups: *P *≤ 0.01. **indicates the significant difference between the Codop I + II and the II (C‐DnaE‐3’N6 CDS) codop groups: *P *≤ 0.01. All *P*‐values are reported in Appendix Table S1.Peptides sequences obtained by LC‐MS analysis which include the N6 splitting point which is correctly reconstituted; S: Ser 962 (*n* = 5; biological replicates). Source data are available online for this figure.

To further improve the efficiency of the N6 split‐inteins, we codon‐optimised the N6 CDS (CodopN6), as this has been previously reported to improve F8 levels (Ward *et al*, 2011; McIntosh *et al*, 2013). A fourfold increase in CodopN6 protein expression and secretion was observed by WB compared with the non‐codon‐optimised N6 split‐inteins (Fig 4A and B). Moreover, cells expressing CodopN6 had higher F8 activity levels (~200 IU/dl) than the corresponding non‐codopN6 (~70 IU/dl) as assessed by chromogenic assay (Fig 4C). In addition, to demonstrate that PTS results in precise CodopN6 reconstitution, we transfected HEK293 cells with the CodopN6 intein plasmids and immunopurified the resultant full‐length N6 protein. Liquid chromatography‐mass spectrometry (LC‐MS) analysis showed reconstituted CodopN6 peptides with sequences at the splitting point, which were identical to full‐length CodopN6 encoded by a single plasmid.

This was confirmed across 5 independent experiments and a total number of 211 individual peptides (Fig 4D).

### Systemic administration of AAV‐N6 intein results in therapeutic levels of F8 in HemaA mice

To determine the efficiency of liver gene therapy following systemic administration of either AAV‐N6 intein (N6 intein), AAV‐CodopN6 intein (CodopN6 intein) or the single AAV‐codon‐optimised *F8*‐V3 (CodopV3; Nathwani *et al*, 2018) used as gold standard, we generated AAV8 in combination with the small hybrid liver promoter (HLP; McIntosh *et al*, 2013).

**Figure 5:**
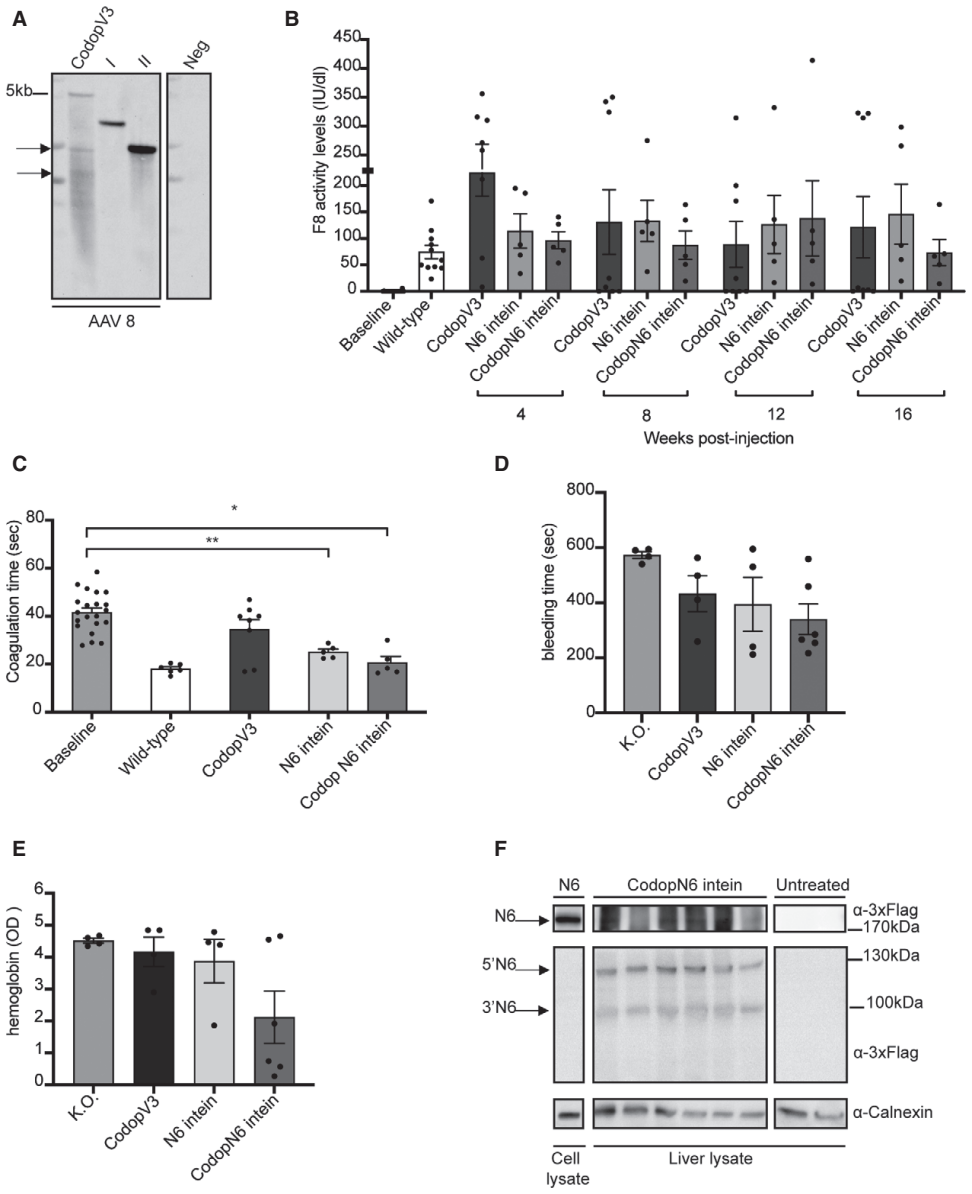
AAV‐N6 intein administration results in therapeutic F8 activity levels Alkaline gel Southern blot analysis of AAV DNA. AAV DNA was hybridised to a probe specific for the HLP promoter. Neg, AAV DNA treated with Dnase I; CodopV3, AAV‐CodopV3; I, AAV‐5’N6‐N‐intein; II, AAV‐C‐intein‐3’ N6.Chromogenic assay performed on plasma samples to detect F8 activity in AAV‐treated mice compared to controls groups. Data are presented as mean ± SEM. The same animals were analysed at the various time points. Each dot within the same group and within different groups of treatment corresponds to a single animal. The baseline includes all animals (*n* = 20) before the treatment: CodopV3: *n* = 8; N6 intein: *n* = 5; CodopN6 intein: *n* = 5. The statistical difference between groups has been assessed at 4‐week post‐injection (wpi) with the one‐way ANOVA *P* = 0.056; at 8 wpi with the Kruskal–Wallis test *P *= 0.68; at 12 wpi with the Kruskal–Wallis test *P* = 0.45; at 16 wpi with the Kruskal–Wallis test *P* = 0.58. All *P*‐values are reported in Appendix Table S1.Activated partial thromboplastin time (aPTT) assay performed on plasma samples both at the baseline and at the last time point of the analysis (16 wpi). Data are presented as mean ± SEM. Each dot within each group of treatment corresponds to different animals: The baseline includes all animals before the treatment *n* = 21; CodopV3: *n* = 8; N6 intein: *n* = 5; CodopN6 intein: *n* = 5. Significant differences between groups were assessed using the Kruskal–Wallis test. **indicates the significant difference between the baseline and the N6 intein groups: *P *≤ 0.01.*indicates the significant difference between the baseline and the CodopN6 intein groups: *P *≤ 0.05. All *P*‐values are reported in Appendix Table S1.Tail‐clip assay reported as bleeding time in seconds. Significant differences between groups were assessed using one‐way ANOVA test *P*‐value is = 0.11. Data are presented as mean ± SEM. Each dot corresponds to a different animal. Knockout (K.O.) group: *n* = 4; CodopV3: *n* = 4; N6 intein: *n* = 4; CodopN6 intein: *n* = 6.Haemoglobin content measured as optical density (OD) at 416 nm after the tail‐clip assay. Significant differences between groups were assessed using one‐way ANOVA test. Data are reported as mean ± SEM. Each dot corresponds to a single animal. Knockout (K.O.) group: *n* = 4; CodopV3: *n* = 4; N6 intein: *n* = 4; CodopN6 intein: *n* = 6.Western blot analysis of liver lysates (100 µg) from either CodopN6 intein‐treated mice (*n* = 6) or untreated haemophilic mice (*n* = 2). A lysate from HEK293 cells transfected with the N6 full‐length plasmid was used as positive control (50 µg). Arrows point at N6 full‐length protein (N6), the 5’ half of N6 (5’N6) and 3’ half of N6 (3’N6). Source data are available online for this figure.

The resulting size of both the N6 intein and CodopN6 intein AAVs genomes (3.7 kb for the 5’half and 3 kb for the 3’half) fell well within the AAV packaging capacity, unlike the genome of the single AAV‐CodopV3 (5.2 kb) which exceeds the capacity (Nathwani *et al*, 2018). The packaged genomic integrity of the 3’ and 5’ AAV‐N6 intein was confirmed by alkaline Southern blot hybridisation of purified vector DNA with a probe specific for the HLP promoter. The lanes corresponding to AAV‐intein vectors showed discrete bands of the expected molecular weight while the lane corresponding to the AAV8‐Codop‐V3 vector consisted of a heterogeneous population of truncated genomes of different sizes (Fig 5A).

The AAV‐N6 intein vectors and the single AAV‐CodopV3 were injected retro‐orbitally in 7‐11‐week‐old adults HemA ^B6;129S‐F8tm1Kaz^ mice, at a dose of 5 × 10^11^ genome copies (GC) of each vector per animal. Since the N6 intein codon optimisation allows higher levels of both F8 activity and expression *in vitro*, we hypothesised that this would allow the AAV vector dose to be lowered to obtain similar therapeutic efficacy to the non‐codon‐optimised N6. For this reason, the AAV‐CodopN6 intein set was administered at the dose of 1.5 × 10^11^genome copies of each vector. Blood plasma samples were collected for 16 weeks following vector administration at 4‐week intervals. F8 activity was monitored using both the functional chromogenic assay and the activated partial thromboplastin time (aPTT).

After AAV‐N6 intein administration, plasma F8 activity reached wild‐type levels (~150 IU/dl) and remained stable up to 16‐week post‐injection (wpi). These mean levels were similar to those from animals which received the single AAV‐CodopV3 (Fig 5B), which however showed a significant decrease or total loss of F8 activity over time in the majority of the treated animals (5 out of 8, Fig 5B).

Mice injected with the AAV‐CodopN6 intein showed wild‐type mean levels of F8 activity comparable to those obtained with the AAV‐N6 intein vectors up to 12 wpi. A slight decrease in F8 activity levels was observed at 16 wpi in some of the animals (2 out of 5, Fig 5B), although the average levels were still in the wild‐type range (Fig 5B). To confirm that the levels of F8 activity obtained resulted in improved blood clotting, we measured the activated partial thromboplastin time (aPTT) in mouse plasma at the time point of 16‐week post‐injection after AAV vector administration. We found that this was significantly decreased to normal levels in animals which received either the AAV‐N6 intein or the AAV‐CodopN6 intein treatment (Fig 5C). Moreover, to assess the possible correction of the haemophilic phenotype in the AAV‐treated mice compared with knockout untreated controls, we performed the tail‐clip assay. For this purpose, a new round of injections was performed; three groups of animals were injected as previously described, and the assay was carried out at 4–16‐week post‐injection. All the groups of AAV‐treated mice showed a trend in reduction of bleeding time (Fig 5D) and of blood loss (Fig 5E) compared with haemophilic untreated controls.

To demonstrate that transduced hepatocytes express the N6 full‐length protein, we performed Western blot analysis on liver lysate samples from the AAV‐CodopN6 intein injected group (Fig 5F), which revealed a band of the expected molecular weight.

### Mice injected with AAV‐CodopV3 develop anti‐F8 antibodies

**Figure 6:**
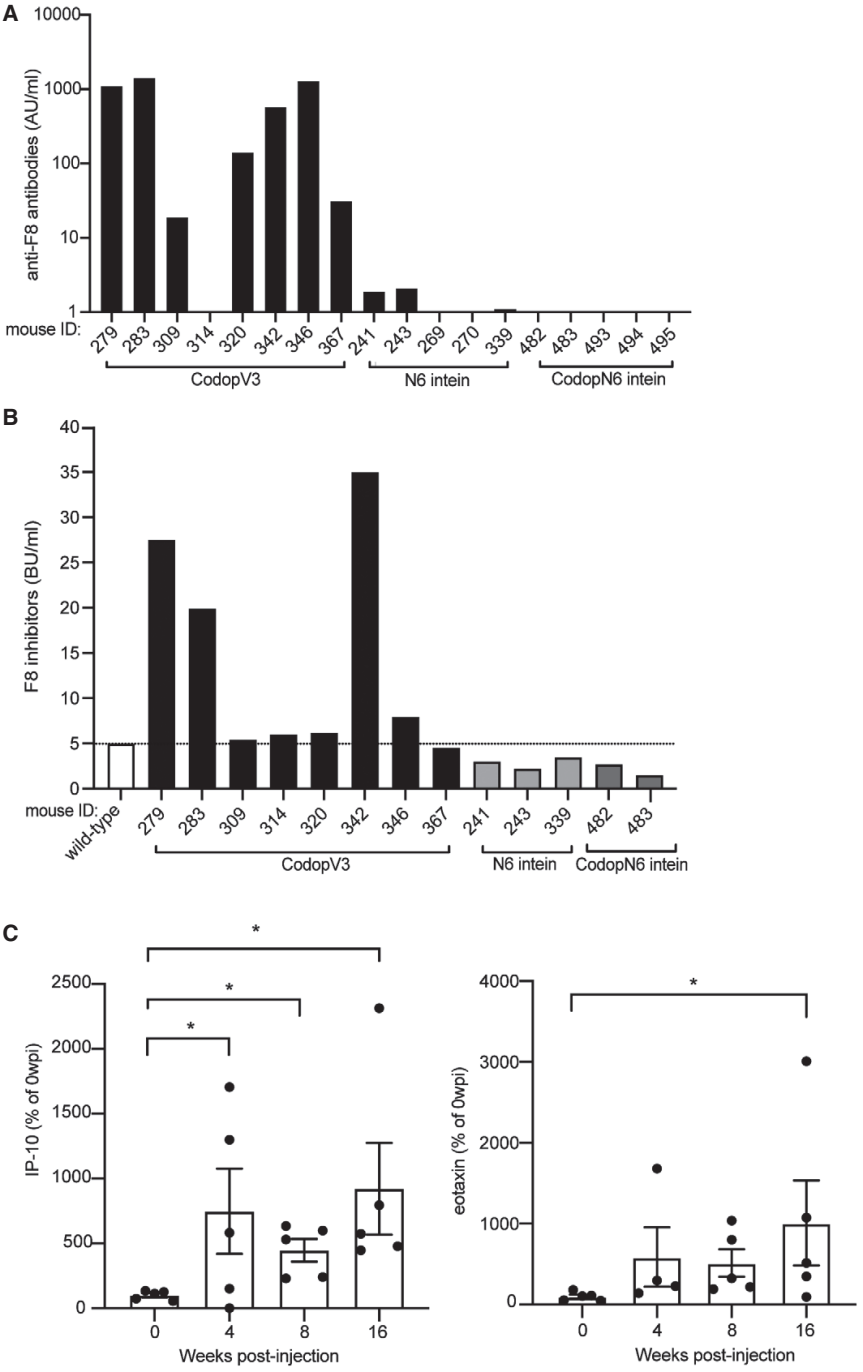
AAV‐N6 intein administration results in therapeutic F8 levels without eliciting anti‐F8 antibodies The amount of anti‐F8 antibodies analysed by indirect ELISA is reported in Arbitrary Units/millilitre (AU/ml). Each numbered bar represents a single mouse. CodopV3, AAV‐CodopV3 injected group *n* = 8; N6 intein, AAV‐N6 intein injected group *n* = 5; CodopN6 intein, AAV‐CodopN6 intein injected group *n* = 5. Significant differences were assessed as follow: Paired *T*‐test has been used for CodopV3 *n* = 5: *P *≤ 0.001; Wilcoxon test for CodopV3 *n* = 3: *P *≤ 0.05; Paired *T*‐test has been used for N6 intein *n* = 5: *P* ≤ 0.05; Paired *T*‐test has been used for CodopN6 intein *n* = 5 *P* ≤ 0.01.Bethesda assay performed on plasma samples mice injected with AAV‐CodopV3; on the plasma of animals injected with AAV‐N6 intein *n* = 3 and the plasma of animals injected with AAV‐CodopN6 intein *n* = 2; plasma of a wild‐type animal was used as a control. The dotted line indicates the threshold above which anti‐F8 antibodies are considered inhibitors.Pro‐inflammatory cytokines levels followed over time and reported as percentage of the baseline levels (time point before the injection). Data are represented as mean ± SEM. Five animals belonging to the CodopV3 group were analysed. Significant differences were assessed using the Kruskal−Wallis test; for IP‐10: *indicates the *P *≤ 0.05 between 0 and 4 wpi; * indicates the *P *≤ 0.05 between 0 and 8 wpi; *indicates the *P *≤ 0.05 between 0 and 16 wpi. (*n* = 20); for eotaxin: *indicates the *P *≤ 0.05. All *P*‐values are reported in Appendix Table S1. Source data are available online for this figure.

We evaluated anti‐F8 antibodies development in AAV‐treated animals: serum from animals administered with the single AAV‐CodopV3 (*n* = 8), the AAV‐N6 intein (*n* = 5) and the AAV‐CodopN6 intein (*n* = 5) were analysed by an indirect enzyme‐linked immunosorbent assay (ELISA). We found that anti‐F8 antibody levels were precisely inversely correlated with the F8 activity. Specifically, of the *n* = 8 animals injected with the single AAV‐CodopV3, *n* = 5 had high levels of anti‐F8 antibodies and no detectable F8 activity (*P < 0.05) while the mice exhibiting high F8 activity levels (*n* = 3) had no anti‐F8 antibodies (***P < 0.0008) (Fig 6A). All mice injected with AAV‐N6 intein (*n* = 5) or with AAV‐CodopN6 intein (*n* = 5) had high F8 activity levels and no detectable anti‐F8 antibodies *P < 0.05 and **P < 0.005, respectively. We further investigated the possible inhibitory activity of the anti‐F8 antibodies. The Bethesda assay performed on plasma samples of mice injected with the single AAV‐CodopV3 with high levels of anti‐F8 antibodies measured by indirect ELISA, confirmed the inhibitory activity of the anti‐F8 antibodies in this group (Fig 6B).

To characterise immune responses to F8 variants in AAV‐injected mice, we analysed their serum cytokines levels both at baseline and at 4‐, 8‐ and 16‐weeks post‐injection. Mice injected with the single AAV‐CodopV3 had significantly higher levels of the two pro‐inflammatory cytokines eotaxin and interferon‐gamma‐induced protein 10 (IP‐10) (Rosenkilde & Schwartz, 2004; Boström *et al*, 2015; Li *et al*, 2021) than animals injected with AAV‐intein vectors (Fig 6C). Since neither pro‐ nor anti‐inflammatory cytokines, such as IL‐10 and TGF‐ß among those tested, were increased in the AAV‐N6 intein‐treated animals, N6 appears rather immunologically inert at the vector doses tested when compared to V3.

## Discussion

After decades of extensive research on therapies for haemophilia A (HemA), numerous new technologies have emerged offering a broader range of disease management options for HemA patients. Yet, the problem of curing the disease remains unsolved. Liver gene therapy with single AAV vectors has the potential to fill this gap and is currently under evaluation in multiple clinical trials (Butterfield *et al*, 2019; Makris, 2020). However, the high F8 protein levels initially observed in some of the trials require high doses of viral vectors to be achieved (Makris, 2020). In addition, the durability of vector expression has recently been questioned because of the F8 declining levels observed in the gene therapy trial *NCT02576795* (Pasi *et al*, 2020). Thus, we are still in the early stages of developing viable gene therapy for HemA and further improvements will be required to obtain safe and sustained F8 expression from human liver.

Here, we show that dual AAV vectors armed with *Npu* DnaE split‐inteins efficiently and precisely reconstitute the large and highly secreted F8‐N6 (N6) variant in the mouse liver resulting in stable therapeutic levels of F8.

The N6 variant has been previously delivered in mice in the context of a single AAV, but its size (5 Kb) greatly exceeds the normal AAV cargo capacity. This construct was shown to achieve high levels of F8 expression and secretion (McIntosh *et al*, 2013), but clinical translatability is limited by the poor characterisation of oversize genomes upon truncated genome re‐assembly. In this study, we overcome this limitation by effectively delivering N6 using two separate AAV vectors, each well within the AAV packaging capacity.

The therapeutic levels of F8 achieved *in vivo* by a single systemic administration of AAV‐N6 intein are comparable to those obtained with the single packageable AAV‐codon‐optimised *F8*‐V3 (CodopV3; Nathwani *et al*, 2018) which is in clinical development (*NCT03001830*) and expresses one of the most promising B‐domain deleted (BDD) versions of F8 (Nathwani *et al*, 2018). Yet, the size of the AAV‐*F8*‐CodopV3 genome is over the canonical vector cargo capacity and results in truncated genomes.

Importantly, we provide evidence that our strategy does not lead neither to the development of anti‐F8 antibodies or to increased levels of pro/anti‐inflammatory cytokines, whereas this occurred in AAV‐CodopV3 treated animals. There could be several reasons for this: while the CodopV3 coding sequence (CDS) is fully contained within the longer N6 CDS, it is possible that the difference in their B‐domain structure is responsible for the higher immune‐reactivity of V3 than N6; F8 antigen levels may differ between the various groups, as we engineered N6 to be expressed at similar therapeutic levels than V3 using lower vector doses, which improves the burden of both AAV manufacturing and anti‐AAV immune responses; the single oversized AAV‐CodopV3 vector contains truncated genomes that may result in shorter immunogenic products.

One of the limitations of the AAV‐intein platform is the production of half proteins derived from non‐*trans*‐spliced polypeptides (for instance, in cells infected by only one of the two AAV‐intein vectors) as well as of the inteins excised from the mature protein, which is an expected by‐product of PTS. Reports of intein‐mediated *trans*‐splicing also occurring in the cell medium of independently expressed and secreted intein halves (Zhu *et al*, 2010, 2011) suggest that this could contribute to the overall F8 levels even if not all cells are coinfected. The excised inteins bioproduct could raise immunological concerns as it is non‐mammalian; however, no apparent toxicity has been observed in previous studies (Chen *et al*, 2007; Zhu *et al*, 2010, 2011, 2013; Tornabene *et al*, 2019) nor in ours, though our study was not designed to assess potential toxicity. Independently, we have recently incorporated a degron in the AAV‐intein vectors that results in selective intein degradation following PTS, therefore without significantly impacting on levels of full‐length protein (Tornabene *et al*, 2021). This could be incorporated in the future in the AAV‐N6 intein vectors, if necessary.

In conclusion, our results support liver gene therapy with a single intravenous administration of AAV‐N6 intein as a potential therapeutic strategy for HemA.

## Materials and Methods

### Study design

This study was designed to define the efficiency of AAV‐intein–mediated protein *trans*‐splicing (PTS) in reconstituting the full‐length F8‐N6 protein in mouse liver. This was defined *in vitro* by assessing the expression (Western blot) and the activity (chromogenic assay) of the reconstituted protein achieved via protein *trans*‐splicing and *in vivo*. To initially evaluate PTS efficiency in liver, a proof‐of‐concept study was performed in wild‐type mice where we compared AAV‐intein (*n* = 5) to single AAV vectors (*n* = 5) using the enhanced green fluorescent protein (eGFP) as reporter. The AAV‐N6 intein platform was tested in an adult mouse model of haemophilia A in comparison with the single AAV‐codon‐optimised BDD *F8*‐V3 used as golden standard (chromogenic assay, activated partial thromboplastin time and Western blot). In the *in vivo* studies that involved the haemophilic model, males only were used (given that haemophilia A is inherited as X‐linked recessive). To evaluate the efficacy of the treatment overtime, different time points were selected for the analysis as indicated in the results section.

### Statistical analysis

The experimenters in the efficacy studies were blind to the treatment of the animals, and within the same litter, animals were randomly assigned to each treatment group. Sample sizes were determined on the basis of previous experience and technical feasibility; at least three biological replicates in *in vitro* studies or four animals per group were used in all the experiments, as indicated in the results section and figure legends.

Data are presented as mean ± SEM, and statistical *P *≤ 0.05 were considered significant. The normality assumption was verified using the Shapiro–Wilk test. Levene’s test was applied to check the homogeneity of variances. Data were analysed by the Student’s *t*‐test, ANOVA test or, when data were not normally distributed (Shapiro–Wilk test *P *≤ 0.05), either the Kruskal–Wallis rank‐sum test or the Wilcoxon rank‐sum test (non‐parametric tests) were used. Specific statistical values are reported in Appendix Table S1.

### Generation of AAV vector plasmids

The plasmids used for AAV vector production are derived from the pTigem AAV plasmid that contains the ITRs of AAV serotype 2. The enhanced green fluorescent protein (eGFP) coding sequence was split at Cysteine71, while the F8‐N6 protein was split at Serin 962 (Ser962) considering the signal peptide exclusively within the N6 linker (in place of the B domain), aiming to preserve the integrity of the other more critical protein domains. Further split considerations were taken into account based on the intrinsic amino acid residue requirements for efficient protein *trans*‐splicing with the *Npu* inteins. In particular, the main prerequisite is the presence of an amino acid containing either a thiol or hydroxyl group (Cysteine, Serine or Threonine) as the first residue in the 3’ half of the coding sequence (Shah *et al*, 2013; Cheriyan *et al*, 2014).

Split‐inteins included in the plasmids were the split‐inteins of DnaE from *Nostoc punctiforme* (*Npu*; Iwai *et al*, 2006). The plasmids used in the study were under the control of either the ubiquitous cytomegalovirus (CMV) promoter (Tornabene *et al*, 2019) or the liver‐specific hybrid liver promoter (HLP) (McIntosh *et al*, 2013) or the human thyroxin binding globulin (TBG) promoter (Yan *et al*, 2012). The polyadenylation signal (polyA) used was either the short synthetic polyA (Levitt *et al*, 1989) or the bovine growth hormone (BGH) polyA signal. For the generation of the heterologous split‐inteins, the same splitting point (Ser962) was used. The N‐split‐intein flanking the 5’ half plasmid was the N‐intein of DnaB from *Rhodothermus marinus* (*Rma*; Zhu *et al*, 2013; Tornabene *et al*, 2019), while the C‐split‐intein flanking the 3’ half plasmid was the C‐intein of the DnaE. Heterologous split‐intein plasmids were produced under the control of the ubiquitous cytomegalovirus (CMV) promoter since they were only used for *in vitro* purposes. The codon‐optimised BDD F8‐V3 plasmid used as golden standard was kindly provided by Dr Amit C. Nathwani (McIntosh *et al*, 2013).

### AAV vector production and characterisation

Adeno‐associated viral vectors were produced by InnovaVector srl (Pozzuoli, Napoli, Italy) by triple transfection of HEK293 cells as already described (Doria *et al*, 2013). No differences in vector yields were observed between AAV vectors, which include the split‐intein sequences or not.

### Southern blot analyses of AAV vector DNA

DNA was extracted from 6 × 10^10^ viral particles measured as genome copies (GC). To digest unpackaged genomes, the vector solution was incubated with 30 μl of DNase I (04536282001; Roche, Italy) in a total volume of 300 μl, containing 50 mM Tris pH 7.5, and 1 mM MgCl_2_ for 2 h at 37°C. The DNase was then inactivated with 50 mM EDTA, followed by incubation at 50°C for 1 h with proteinase K and 2.5% *N*‐lauryl‐sarcosil solution to lyse the capsids. The DNA was extracted twice with phenol‐chloroform and precipitated with 2 volumes of ethanol 100% and 10% sodium acetate (3 M) and 1 μl of Glycogen (1090139300; Roche, Italy) was performed as previously described (Sambrook & Russell, 2001). Single‐stranded DNA was quantified with Qubit^®^ ssDNA Kit (Q10212; ThermoFisher Scientific, Germany). A probe specific for the HLP promoter was used; 1.4 × 10^10^ GC for the single vector AAV‐Codop V3 and for both the 5’ and the 3’ AAV‐N6 intein were loaded on an alkaline agarose gel electrophoresis (Fig 5A).

### Transfection of HEK293 cells

HEK293 cells were maintained and transfected using the calcium phosphate method (1 μg of each plasmid/well in 6‐well plate format) as already described (Maddalena *et al*, 2018). The total amount of DNA transfected in each well was kept equal by the addition of a scramble plasmid when necessary. 12‐hour post‐transfection (hpt) medium was switched to Opti‐MEM reduced serum medium (31985062; Gibco, ThermoFisher Scientific, Germany) 1 ml/well until the time point of 72 hpt when cells were harvested and medium was collected.

### Western blot analysis

Samples (HEK293 cells or liver lysates) were lysed in RIPA buffer to extract F8 protein. Lysis buffer was supplemented with protease inhibitors (11697498001; Roche, Basel, Switzerland) and 1 mM phenylmethylsulfonyl. For medium samples, upon cell harvesting at 72‐hour post‐transfection (hpt) medium samples were centrifugated at 4°C for 15 min to remove cell debris. Purified medium was collected; 30 μl of medium mixed with 1× Laemmli sample buffer. All samples were denatured at 99°C for 5 min in 1× Laemmli sample buffer. Liver lysates (100 µg), cell lysates (50 µg) and medium samples were separated by either 12% (for excised intein detection) or 6% (for full‐length F8 protein and single halves detection) SDS–polyacrylamide gel electrophoresis (SDS–PAGE). The antibodies used for immuno‐blotting are as follows: anti‐3xFlag (Dilution 1:2,000; A8592; Sigma‐Aldrich, Saint Louis, MO, USA) to detect the full‐length F8‐N6 protein and both the 5’ and the 3’ halves; anti‐β‐Actin (Dilution 1:1,000; NB600‐501; Novus Biological LLC, Littleton, CO, USA) to detect β‐Actin proteins which were used as loading controls for the 12% SDS–PAGE; anti‐Calnexin (Dilution 1:2,000; ADI‐SPA‐860; Enzo Life Sciences Inc, New York, NY, USA) to detect Calnexin, used as loading controls for the 6% SDS–PAGE. The quantification of the full‐length F8‐N6 bands detected by Western blot was performed using ImageJ software (free download is available at http://rsbweb.nih.gov/ij/).

### Immunoprecipitation and mass spectrometry analysis

Cells were plated in 100 mm plates (5 × 10^5^ cells/plates) and transfected with either the single CodopN6 plasmid or CodopN6 intein plasmids using the calcium phosphate method (20 μg of each plasmid/plate). Cells were harvested 72 hpt, and both the single CodopN6 and the CodopN6 intein proteins were immunoprecipitated using anti‐flag M2 magnetic beads (M8823; Sigma‐Aldrich), according to the manufacturer instructions. Proteins were eluted from the beads by incubation for 15 min in sample buffer supplemented with 4 M urea at 37°C and 10 min at 99°C. Samples were then loaded on a gradient 4–10% SDS–polyacrylamide gel electrophoresis. In total, 8 protein bands (from HEK293 cells transfected 5 times independently with CodopN6 intein plasmids) were cut after staining with Instant Blue (ISB1L; Sigma‐Aldrich) and were used for protein sequencing. Briefly, 8 gel slides were used for digestion by the following enzymes: Lysin and Trypsin. The resulting peptides were identified using nanoscale liquid chromatography coupled to tandem mass spectrometry (nano‐LC‐MS/MS) analysis. Data obtained were processed using MaxQuant and the implemented Andromeda search engine.

### Animal model

Animals were housed at the TIGEM animal facility (Pozzuoli, Italy). C57Bl/6 mice used in the proof‐of‐concept study with the reporter enhanced green fluorescent protein were purchased from Envigo.

The haemophilic mouse model (B6;129S‐F8tm1Kaz/J) was imported from The Jackson Laboratory (JAX stock). Mice were maintained by crossing knockout homozygous females with knockout hemizygous males.

### Retro‐orbital injection of AAV vectors in mice

All procedures on mice were approved from the Italian Ministry of Health; department of Public Health, Animal Health, Nutrition and Food Safety number 379/2019‐PR.

Adult C57Bl/6 mice (5 weeks of age) were retro‐orbitally injected with AAV8 at the dose of 5 × 10^11^ GC of each vector per animal.

Adult knockout males (between 7 and 11 weeks of age) were retro‐orbitally injected with AAV8 with either AAV‐N6 intein or the single AAV‐CodopV3 as a positive control at the dose of 5 × 10^11^ GC of each vector per animal.

AAV‐CodopN6 intein were retro‐orbitally injected with the dose of 1.5 × 10^11^GC of each vector per animal.

### Plasma collection and F8 assays

Briefly, nine parts of blood were collected by retro‐orbital withdrawal into one part of buffered trisodium citrate 0.109 M (5T31.363048; BD, Franklin Lakes, NJ, USA). Blood plasma was collected after samples centrifugation at 3,000 rpm at 4°C for 15 min.

To evaluate F8 activity, chromogenic assay was performed on plasma samples using a Coatest^®^ SP4 FVIII‐kit (K824094; Chromogenix, Werfen, Milan, Italy) according to the manufacturer’s instructions. Standard curve was generated by serial dilution of commercial human F8 (Refacto, Pfizer). Results are expressed as International Units (IU) per decilitre (dl).

Activated partial thromboplastin time (aPTT) was measured on plasma samples with Coatron M4 (Teco, Bünde, Germany) using the aPTT programme following the manufacturer’s manual.

To quantify F8 antigen levels, the ELISA kit (FVIII‐AG; VisuLize FVIII ELISA kit, Affinity Biologicals, Arcore, Italy) was used according to the manufacturer’s instructions.

### Tail‐clip assay

Mice were anaesthetised, and the distal part of the tails was cut at 2–3 mm of diameter and immediately put in a prewarmed 0.9% saline solution and allowed to bleed for 10 min without disturbance. Tails were then cauterised, and mice were sacrificed. The mixture of collected blood and physiological saline solution was centrifuged at 1,500 *g* for 5 min. The collected erythrocytes were lysed with water, and the haemoglobin content was measured at an optical density of 416 nm.

### Indirect enzyme‐linked immunosorbent assay (ELISA) to detect anti‐F8 antibodies

To evaluate the presence of anti‐F8 antibodies, an indirect enzyme‐linked immunosorbent assay (ELISA) was performed using ZYMUTEST™Anti‐VIII Monostrip IgG (RK039A; HYPHEN BioMed, France) according to the manufacturer’s instructions. The secondary antibody used to detect mouse IgG is the goat‐anti‐mouse IgG (H + L) HRP conjugate (Dilution 1:3000; AP308P; Sigma‐Aldrich).

### Bethesda assay to detect F8 inhibitors

Plasma samples containing high levels of F8 were heat inactivated at 58°C for 90 min. A standard curve was generated by serial dilution of the commercial human F8 (Refacto, Pfizer) in F8‐deficient plasma. Serial dilutions of all samples were done in F8‐deficient plasma and then mixed 1:1 with 100% Refacto (Pfizer) diluted in F8‐deficient plasma. All experimental samples and controls were then incubated at 37°C for 2 h. After that, all samples and the standard curve were analysed by aPTT following the manufacturer’s manual.

### Cytokines and chemokines assay

Serum samples were analysed with mouse cytokine & chemokine 36‐Plex ProcartaPlex 1A Panel (EPX360‐26092‐901; ThermoFisher) according to the manufacturer’s instructions.

## Author contributions

**Federica Esposito:** Conceptualization; Data curation; Formal analysis; Supervision; Validation; Investigation; Visualization; Methodology; Writing—original draft; Project administration; Writing—review and editing. **Hristiana Lyubenova:** Data curation; Investigation; Visualization; Methodology; Writing—review and editing. **Patrizia Tornabene:** Investigation; Methodology; Writing—review and editing. **Stefano Auricchio:** Validation; Investigation; Writing—review and editing. **Antonella Iuliano:** Formal analysis; Writing—review and editing. **Edoardo Nusco:** Resources; Investigation; Writing—review and editing. **Simone Merlin:** Resources; Supervision; Writing—review and editing. **Cristina Olgasi:** Resources; Supervision; Writing—review and editing. **Marco Gargaro:** Resources; Supervision; Writing—review and editing. **Giorgia Manni:** Data curation; Investigation; Writing—review and editing. **Francesca Fallarino:** Resources; Supervision; Writing—review and editing. **Antonia Follenzi:** Resources; Supervision; Writing—review and editing. **Alberto Auricchio:** Conceptualization; Resources; Supervision; Funding acquisition; Methodology; Writing—original draft; Project administration; Writing—review and editing.

In addition to the CRediT author contributions listed above, the contributions in detail are:

The study was conceived, designed and written by AA and FE All data were generated by FE and HL with the technical help of SA. HL and FE performed the *in vitro* experiments, and FE performed the *in vivo* experiments in HemA mice. HL and PT performed the *in vivo* experiments in wild‐type mice with eGFP. AI performed statistical analysis. EN performed the experimental procedures in the animals; SM and CO supervised the technical set‐up of the experiments. PT contributed to the editing of the manuscript. AF, PT and FF contributed to data analysis and interpretation. MG and GM performed cytokines and chemokines analysis.

## Disclosure and competing interests statement

A.A., F.E. and H.L. are coinventors on the patent application number EP201697125. AA. and P.T. are coinventors on the patent application WO2020079034A2. Alberto Auricchio is an editorial advisory board member. This has no bearing on the editorial consideration of this article for publication. The other authors declare that they have no conflict of interest.

## Supporting information



Appendix

Expanded View Figures PDF

Source Data for Expanded View

Source Data for Figure 1

Source Data for Figure 2

Source Data for Figure 3

Source Data for Figure 4

Source Data for Figure 5

Source Data for Figure 6

## Data Availability

All data associated with this study are present in the paper or in the Expanded View. The mass spectrometry proteomics data have been deposited to the ProteomeXchange Consortium via the PRIDE (Perez‐Riverol *et al*, 2022) partner repository and are available in the following database (http://www.ebi.ac.uk/pride/archive/projects/PXD031884). Mass spectrometry proteomics data: identifier PXD031884.
