# Supplementary material for: Liver gene therapy with intein‐mediated F8 trans‐splicing corrects mouse haemophilia A
Source: EMBO Mol Med. 2022 May 2;14(6):e15199. doi: 10.15252/emmm.202115199 (PMC9174883; doi:10.15252/emmm.202115199)
Supplement: Supplementary file 1 — Appendix [file EMMM-14-e15199-s004.pdf]

Appendix

The appendix contains Appendix Table S1 which includes the statistical tests used and p-values

Table of content

Appendix Table S1

Appendix Table S1

Figure 2C The Kruskal-Wallis test followed by the posthoc analysis: Nemenyi's All-Pairs Rank Comparison Test.
The Kruskal-Wallis test p value = 2.88e-05. Post-hoc p values are as follows:

Group 1	Group 2	p value
wild-type F8	Neg	0.8
N6	Neg	0.00034878
SQ	Neg	0.09
V3	Neg	0.002
N6	wild-type	0.003
SQ	wild-type	0.46
V3	wild-type	0.02
SQ	N6	0.3
V3	N6	0.9
V3	SQ	0.6

Figure 3C The Kruskal-Wallis test p value= 0.013.
P value between different groups as follow:

Group 1	Group 2	p value
I	II	0.5
I	I N-int B	0.6
II	I+II (Het)	0.7
II	N6 intein	0.09
II	Neg	0.06
I	I (N int-B)	0.3
II	I+II (Het)	0.4
II	I+II	0.02
II	Neg	0.2
I (N int-B)	I+II (Het)	0.8
I (N int-B)	I+II	0.2
I (N int-B)	neg	0.02
I+II (Het)	I+II	0.1
I+II (Het)	Neg	0.03
I+II	Neg	0.0003

Figure 4C The Kruskal-Wallis test has been used; p value is 0.027.
P value between different groups as follow:

Group 1	Group 2	p value
I	II	0.5
I	Codop II	0.9
I	Codop I	0.9
II	Codop I+II	0.007
II	I+II	0.03
II	Neg	0.4
I	Codop II	0.6
I	Codop I	0.5
I	Codop I+II	0.03
I	I+II	0.1
I	Neg	0.8

II Codop	Codop I+II	0.008
II Codop	I+II	0.04
II Codop	Neg	0.4
I Codop	Codop I+II	0.005
I Codop	I+II	0.03
I Codop	Neg	0.4
Codop I+II	I+II	0.5
Codop I+II	Neg	0.056

Figure 5B The Kruskal-Wallis test has been used at the baseline; p value is 0.0001.
P value between different groups are as follows:

Group 1	Group 2	p value
wild-type	CodopN6 intein	0.0016
wild-type	CodopV3	0.0012
wild-type	N6 intein	0.0016
CodopN6 intein	CodopV3	1
CodopN6 intein	N6 intein	1
CodopN6 intein	CodopV3	0.078

Figure 5B at 4wpi the One-way Anova has been used; p value = 0.056.
P value between different groups are as follows:

Group 1	Group 2	p value
CodopN6 intein	CodopV3	0.078
CodopN6 intein	N6 intein	0.95
CodopV3	N6 intein	0.138

Figure 5B 8wpi the Kruskal-Wallis test has been used; p value is 0.68.
P value between different groups are as follows:

Group 1	Group 2	p value
CodopN6 intein	CodopV3	1
CodopN6 intein	N6 intein	1
CodopV3	N6 intein	1

Figure 5B at 12wpi the Kruskal-Wallis test has been used; p value is 0.45.
P value between different groups are as follows:

Group 1	Group 2	p value
CodopN6 intein	CodopV3	0.84
CodopN6 intein	N6 intein	1
CodopV3	N6 intein	0.94

Figure 5B at 16wpi the Kruskal-Wallis test has been used; p value is 0.58.
P value between different groups are as follows:

Group 1	Group 2	p value
CodopN6 intein	N6 intein	0.90

Figure 5C The Kruskal-Wallis test has been used; p value is <0.0001.
P value between different groups are as follows:

Group 1	Group 2	p value
baseline	CodopN6 intein	0.01
baseline	N6 intein	0.008
baseline	CodopV3	0.7
baseline	wild-type	0.002
CodopN6 intein	CodopV3	0.45
CodopN6 intein	N6 intein	1

CodopN6 intein	wild-type	1
CodopV3	CodopN6 intein	1
CodopV3	wild-type	0.5

Figure 5D Anova test has been used; p value is =0.11.
P value between different groups are as follows:

Group 1	Group 2	p value
CodopN6 intein	CodopV3	0.724
CodopN6 intein	K.O.	0.081
CodopN6 intein	N6 intein	0.926
CodopV3	K.O.	0.492
CodopV3	N6 intein	0.978
K.O.	N6 intein	0.294

Figure 5E Anova test has been used; p value is =0.39.
P value between different groups are as follows:

Group 1	Group 2	p value
CodopN6 intein	CodopV3	0.161
CodopN6 intein	K.O.	0.085
CodopN6 intein	N6 intein	0.265
CodopV3	K.O.	0.986
CodopV3	N6 intein	0.991
K.O.	N6 intein	0.920

Figure 6A Paired T-test has been used
P value between different groups are as follows:

Test	Group	p value
Paired T-test	CodopV3	0.00072
Paired T-test	N6 intein	0.033
Paired T-test	CodopN6 intein	0.002

Figure 6C The Kruskal-Wallis test has been used; p value is 0.01140.
P value between different groups are as follows:

Group	Group 1	Group 2	p value
	0	4	0.01623160
	0	8	0.04917713
	0	16	0.01623160
	4	8	0.64377442
	4	16	0.93665725
IP10%	8	16	0.64377442

Figure 6C The Kruskal-Wallis test has been used; p value is 0.03650.
P value between different groups are as follows:

Group	Group 1	Group 2	p value
	0	4	0.09686514
	0	8	0.06366775
	0	16	0.04957186
	4	8	0.84251318
	4	16	0.84251318
eotaxin %	8	16	0.84251318
