# Supplementary material for: Liver gene therapy with intein‐mediated F8 trans‐splicing corrects mouse haemophilia A
Source: EMBO Mol Med. 2022 May 2;14(6):e15199. doi: 10.15252/emmm.202115199 (PMC9174883; doi:10.15252/emmm.202115199)
Supplement: Supplementary file 2 — Expanded View Figures PDF [file EMMM-14-e15199-s008.pdf]

Expanded View Figures

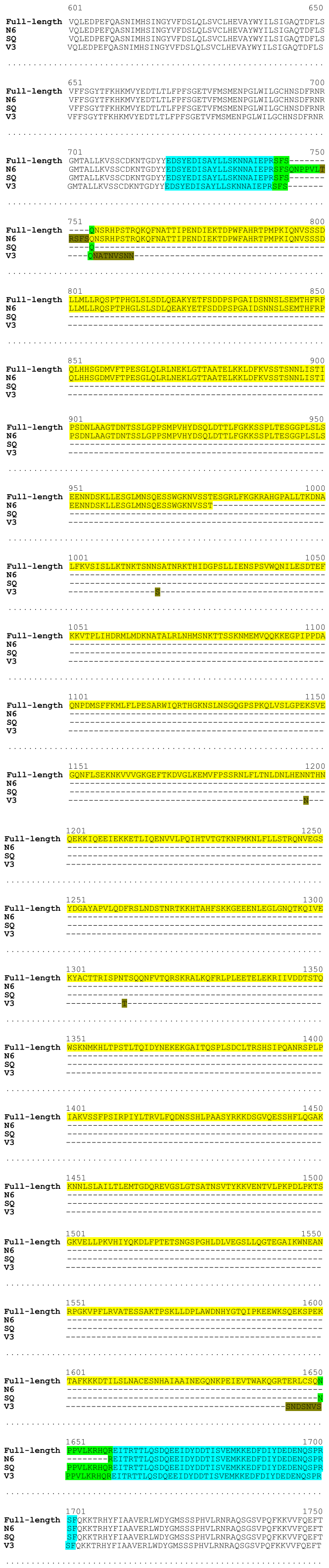

Figure EV1. Amino acid sequence alignment underlining the differences in the B domain of the F8 variants.

The amino acid (aa) sequences of the B domain (or substitution linkers corresponding to it) of the F8 variants were aligned using the Clustal Omega software. a2 acidic linker of the F8 protein spans from aa position 720 to 740; the entire B domain or its substitutions are shown between aa 741 and 1659; a3 acidic linker spans from aa 1660 to 1702. Full-length, wild-type F8; N6, BDD F8-N6; SQ, BDD F8-SQ; V3, BDD F8-V3.

© 2022 The Authors

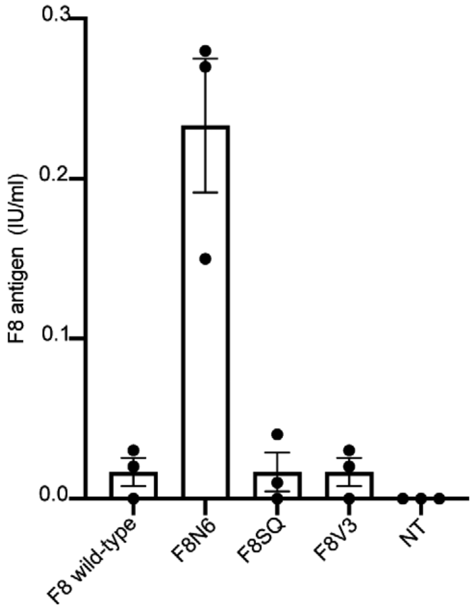

Figure EV2. F8 antigen quantification in the media of transfected HEK293 cells.

Enzyme-linked immunosorbent assay (ELISA) for F8 antigen performed on medium of HEK293 cells transfected with plasmid encoding for each hF8 variant (n = 3 biological replicates). F8 antigen levels are reported as International Units/millilitres (IU/ml). Data are represented as mean ± SEM.

Source data are available online for this figure.
