# Supplementary material for: Liver gene therapy with intein‐mediated F8 trans‐splicing corrects mouse haemophilia A
Source: EMBO Mol Med. 2022 May 2;14(6):e15199. doi: 10.15252/emmm.202115199 (PMC9174883; doi:10.15252/emmm.202115199)
Supplement: Supplementary file 10 [file EMMM-14-e15199-s010.pdf]

EMBO Molecular Medicine

Liver gene therapy with intein-mediated F8 trans-splicing corrects mouse hemophilia A

Federica Esposito, Hristiana Lyubenova, Patrizia Tornabene, Stefano Auricchio, Antonella Iuliano, Edorado Nusco, Simone Merlin, Cristina Olgasi, Marco Gargaro, Giorgia Manni, Francesca Fallarino, Antonia Follenzi, and Alberto Auricchio
DOI: 10.15252/emmm.202115199

Corresponding author: Alberto Auricchio (auricchio@tigem.it)

Review Timeline:

Submission Date:	13th Oct 21
Editorial Decision:	5th Nov 21
Revision Received:	10th Mar 22
Editorial Decision:	28th Mar 22
Revision Received:	1st Apr 22
Accepted:	11th Apr 22

Editor: Zeljko Durdevic

Transaction Report:

(Note: With the exception of the correction of typographical or spelling errors that could be a source of ambiguity, letters and reports are not edited. Depending on transfer agreements, referee reports obtained elsewhere may or may not be included in this compilation. Referee reports are anonymous unless the Referee chooses to sign their reports.)

5th Nov 2021

Dear Prof. Auricchio,

Thank you for the submission of your manuscript to EMBO Molecular Medicine. We have now received feedback from the three reviewers who agreed to evaluate your manuscript. As you will see from the reports below, the referees acknowledge the interest of the study but also raise important and partially overlapping concerns particularly regarding the limited conceptual advance of the study and the lack of the molecular mechanism behind the immune tolerance towards trans-spliced F8.

Taking this in consideration it is clear that publication of the paper cannot be considered at this stage. After our cross-commenting session it became clear that defining the immune mechanism would be necessary for further consideration of the manuscript. I also note that addressing this aspect and the reviewers concerns in full appears to require a lot of additional work and experimentation. I am unsure whether you will be able or willing to address those and return a revised manuscript within the 6 months deadline.

Please note that further consideration of a revision that addresses reviewers' concerns in full will entail a second round of review. EMBO Molecular Medicine encourages a single round of revision only and therefore, acceptance or rejection of the manuscript will depend on the completeness of your responses included in the next, final version of the manuscript. For this reason, and to save you from any frustrations in the end, I would strongly advise against returning an incomplete revision and would also understand your decision if you chose to rather seek rapid publication elsewhere at this stage.

I look forward to seeing a revised form of your manuscript. Use this link to login to the manuscript system and submit your revision: Link Not Available

Should you find that the requested revisions are not feasible within the constraints outlined here and choose, therefore, to submit your paper elsewhere, we would welcome a message to this effect.

Yours sincerely,

Zeljko Durdevic

Zeljko Durdevic
Editor
EMBO Molecular Medicine

***** Reviewer's comments *****

Referee #1 (Comments on Novelty/Model System for Author):

Although there is no true technological and rational novelty in this gene therapy approach, the experiments are well performed and clearly presented and the results straightforward.
This research wok demonstrates that the trans splicing approach would be feasible for gene therapy of Hemophilia A and opens a new prospect for future clinical investigation. Still, it is a bit disappointing that the relevant finding of lack of immune response against the transgene in the present experimental settings is not extensively discussed.

Referee #1 (Remarks for Author):

The manuscript of Esposito and collaborators describes a gene therapy approach for hemophilia A (HemA) based on AAV-intein platform, aimed at overcoming packaging limitations posed by the large F8 coding sequence. The authors took advantage of their consolidated expertise in gene therapy and protein trans splicing to successfully demonstrate that in vivo co-injection of two AAV vectors, each bearing half of the highly active F8-N6 variant cDNA modified by the addition of split inteins, can mediate efficient full length F8 synthesis and correction of the bleeding defect in HemA mouse model.
Currently, a single AAV8 containing a B domain deleted version of F8-6N variant is under clinical investigation and represents the gold standard for AAV-mediated HemA gene therapy. However, this construct still slightly exceeds the loading limit of AAV, with the consequent drawback of generating a library of heterogeneous vectors with truncated genomes. Moreover, the long-term efficiency of gene transfer is restricted by generation of neutralizing anti F8 antibodies in vivo.
Intriguingly, the present findings not only underline the efficiency and therapeutic potential of F8 factor generated by AAV-intein trans splicing, but noteworthy, contrary to the single oversize AAV8-F8N6 vector, this expression platform does not elicit anti-F8 antibodies in the treated animals over the 16 weeks of experimental investigation.

Major comments.

The actual noteworthy finding of this work is the absence of the development of neutralizing antibodies anti F8 in the AAV-intein treated animals. This observation, if confirmed, has great potential to represent a step forward in the treatment of Hemophilia A or similar diseases.
What is missing in the present work is a convincing explanation of the mechanism at the basis of the apparent immune tolerance toward trans spliced F8 protein, a data that deserves more in-depth investigation and discussion.
In this respect:

i) As the authors anticipated, how early phase events following transduction may be critical to the determination of immunological response to the transgenic product?
ii) Does it make sense to speculate on the possible role of the emi polypeptides generated by the split AAV-intein vectors?
iii) Is there any dosage effect of the transgenic product? There is no estimation of the global efficiency of the trans splicing process specifically in the target liver tissue in vivo, and how abundant is the full length F8 protein in comparison with the transgene produced by the single AAV8-F8N6 vector.

Minor comments.

i) What about trans spliced protein is produced in other tissues, considering that good tropism of AAV8 also for muscle?

ii) In WB of Figure 2-B and 2-C and Figure 3-A and 3-B, second panel, arrows and MW can help to clearly identifying relevant protein bands.

Referee #2 (Remarks for Author):

Although currently tested in multiple clinical trials, AAV gene therapy for hemophilia A is complicated by the size of FVIII and the packaging limit of AAV vectors. Esposito et al offer a solution to this problem by engineering of a dual vector expressing FVIII heavy and light chains (of the highly active N6 FVIII variant) fused to intein sequences, so that protein splicing restores the complete molecule. The authors show that correctly sliced B domain-deleted N6-FVIII is produced, which has biological activity. An important finding is that very little antibody is formed against the N6 variant after liver gene transfer in immune competent hemophilia A mice.

While a nice contribution to the field, there are several limitations:

1. Different doses of vector were used for the different variants when compared in vivo, complicating conclusions about efficacy. It does not appear that the intein approach provides much of an improvement in efficacy. Codon optimization dis not really improved intein-N6 in vivo.
2. Efficiency of proteins trans-splicing is unclear. It is not unclear if there is a chain imbalance and how much unspoiled single chains are generated and secreted.
3. The reason for lack of anti-FVIII responses against intein-N6 FVIII is unknown.
4. Cytokine data on serum samples are not informative and should be removed.

Referee #3 (Remarks for Author):

In this manuscript, Esposito and co-workers report on an application of the dual intein system for gene therapy of haemophilia A. The authors take advantage of the natural protein trans-splicing property of trans-inteins to reconstitute the expression and secretion of a large, engineered version of F8 in liver, reaching a therapeutically relevant circulating concentration of this factor. Of interest, this approach did not elicit production of anti-F8 antibodies, which were instead observed in the AAV treatments currently under clinical investigation for haemophilia A.

The manuscript is interesting and most of the results are sound. The demonstration that F8 can be expressed in the liver using AAV vectors to express split portions of F8 through the intein system is interesting in itself for haemophilia A, but can also pave the way to other gene therapy applications with inserts longer than the packaging capability of AAV.

The following is a list of suggestions for improvement.

1) A clear indication of the difference in activity of the wild-type form of F8 compared to the N6, V3 and SQ variants is missing. The authors should specify what they mean with "activity" and better explain such differences.
2) Page 8 line 144. If the activity assay is so variable, how were the authors able to decide which variant was the best? Is there a more reliable way to define the differences among the three variants?
3) Page 5 line 155. The splitting point was chosen within the B-domain. Is this related to the occasionally different spliced products that are generated?
4) Fig 3 A-B. The immunoblotting with the dual vector combination shows a band of the expected size but also other weak but visible bands with different mobility. Are these other spliced products? How could these products interfere with F8 expression?
5) The authors report that treatment with F8-N6 did not elicit the production of anti-F8 antibodies. This is an interesting observation, but lacks a molecular explanation, or, at least, an attempt at it. Could vector oversize (F8-V3) be a determinant for antibody response? This point is important, as this difference is pivotal as compared to other treatments.
6) Can the authors comment on the possible immunogenicity of the half size proteins produced by the dual AAV vectors?
7) The immunoblottings should report the relative molecular weights, as these appear to be important in this context.

Point-by-point answer to the Reviewers comments

Referee #1 (Comments on Novelty/Model System for Author):

Although there is no true technological and rational novelty in this gene therapy approach, the experiments are well performed and clearly presented and the results straightforward.

We thank the Reviewer for the overall positive comments on our manuscript. Regarding the comment on the limited novelty, we would like to underline that we provide the first evidence that AAV-intein are effective in the context of liver gene therapy, and that they reconstitute F8 to therapeutic levels in animal models of HemA. While others have previously attempted at reconstituting F8 with intein, they failed at obtaining full-length protein reconstitution in vivo (Chen et al, 2007). Therefore, our data go well beyond the state-of-the-art.

This research wok demonstrates that the trans splicing approach would be feasible for gene therapy of Hemophilia A and opens a new prospect for future clinical investigation. Still, it is a bit disappointing that the relevant finding of lack of immune response against the transgene in the present experimental settings is not extensively discussed.

We have now expanded the experiments and discussion on the reduced immune responses to F8 in the animals treated with AAV-F8-N6 compared to -F8-V3. The current data point to a pro-inflammatory immune response elicited by V3 but not by N6 which however does not seem to induce anti-inflammatory/immune tolerogenic cytokines.

Referee #1 (Remarks for Author):

The manuscript of Esposito and collaborators describes a gene therapy approach for hemophilia A (HemA) based on AAV-intein platform, aimed at overcoming packaging limitations posed by the large F8 coding sequence. The authors took advantage of their consolidated expertise in gene therapy and protein trans splicing to successfully demonstrate that in vivo co-injection of two AAV vectors, each bearing half of the highly active F8-N6 variant cDNA modified by the addition of split inteins, can mediate efficient full-length F8 synthesis and correction of the bleeding defect in HemA mouse model.
Currently, a single AAV8 containing a B domain deleted version of F8-N6 variant is under clinical investigation and represents the gold standard for AAV-mediated HemA gene therapy. However, this construct still slightly exceeds the loading limit of AAV, with the consequent drawback of generating a library of heterogeneous vectors with truncated genomes. Moreover, the long-term efficiency of gene transfer is restricted by generation of neutralizing anti F8 antibodies in vivo.
Intriguingly, the present findings not only underline the efficiency and therapeutic potential of F8 factor generated by AAV-intein trans splicing, but noteworthy, contrary to the single oversize AAV8-F8N6 vector, this expression platform does not elicit anti-F8 antibodies in the treated animals over the 16 weeks of experimental investigation.

Major comments.

The actual noteworthy finding of this work is the absence of the development of neutralizing antibodies anti F8 in the AAV-intein treated animals. This observation, if confirmed, has great potential to represent a step forward in the treatment of Hemophilia A or similar diseases.

While we agree that the absence of neutralizing antibodies is an important finding it is not the only noteworthy since, as also mentioned above, we demonstrate for the first time the therapeutic efficacy of AAV-intein in liver which greatly expands the potential application of AAV for large gene transfer in vivo. For this reason, we have decided to delete from the title the reference to the absence of neutralizing antibodies as we would like to stress the overall therapeutic potential of AAV-intein in liver.

What is missing in the present work is a convincing explanation of the mechanism at the basis of the apparent immune tolerance toward trans spliced F8 protein, a data that deserves more in-depth investigation and discussion.

We would like to point-out that our new data does not support that F8-N6 gene delivery induces a tolerogenic response or well known anti-inflammatory cytokines but rather that F8-V3 is more immunogenic than F8-N6.
This is what the serum cytokine analysis performed at various time points in treated animals shows. Understanding the mechanism underlying the immunogenicity of F8-V3 goes beyond the scope of this manuscript. However, we have added a paragraph to the Discussion, lines 280-287 where we speculate about the hypotheses behind the F8-V3 immunogenicity.

In this respect:
i) As the authors anticipated, how early phase events following transduction may be critical to the determination of immunological response to the transgenic product?

The serum cytokine analysis, which now includes early time points (4 and 8 weeks post-injection) shows significantly increased levels of the two pro-inflammatory cytokines interferon gamma induced protein 10 (IP-10) and eotaxin in the AAV-CodopV3 but not in the AAV-N6 groups starting at 4 weeks post-injection, a time point at which we also detect high levels of anti-F8 antibodies in the AAV-CodopV3-treated mice. These data have been added to the Results section ***Mice injected with AAV-CodopV3 develop anti-F8 antibodies****, lines 243-250.*

ii) Does it make sense to speculate on the possible role of the emi-polypeptides generated by the split AAV-intein vectors?

In principle emi-polypeptides could modulate immune-responses, however, animals treated with AAV-intein vectors which express both full-length and emi-polypeptides have no detectable increase in either anti-inflammatory/immune-tolerogenic or pro-inflammatory cytokines suggesting that full-length F8-N6 or its emi-polypeptides are rather inert when compared to F8-V3 at the AAV vector doses we have investigated.

iii) Is there any dosage effect of the transgenic product?

It is possible that the transgenic product has a dose-dependent immune effect. The overall objective of this study however was to test the therapeutic potential of AAV-intein-F8 in mice. Towards this, we have designed F8-N6 to be delivered at low vector doses and show that indeed it reaches similar therapeutic efficacy than AAV8-V3 given at higher vector doses therefore reducing both its manufacturing and risk burden. If reducing or increasing F8-N6 by delivering different vector doses changes its immune-profile is definitely interesting and worth investigating in the future when moving forward, however it is beyond the original scope of this manuscript. We have added a comment in the Discussion where we speculate on this dosage effect lines 283-285.

There is no estimation of the global efficiency of the trans splicing process specifically in the target liver tissue in vivo, and how abundant is the full-length F8 protein in comparison with the transgene produced by the single AAV8-F8N6 vector.

It is not possible to make a direct comparison with full-length F8N6 since it is too large to fit into a single AAV8 vector. To address the Reviewer comment we have performed a proof-of-concept study in mouse liver, using the enhanced green fluorescent protein (eGFP) as transgene whose size fits into a single AAV vector. Therefore, the same expression cassette either containing the full-length eGFP coding sequence (CDS) or the same CDS split into two halves flanked by inteins has been packaged into either a single or two AAV8-intein vectors, respectively. Western blot analysis of AAV-treated liver lysates shows that AAV-intein reconstitute 76% of the full-length eGFP protein expressed from the single AAV vector. These data are now included in the Results section ***AAV-intein mediated protein trans-splicing in mouse liver****, lines 90-104***.**

Minor comments.

i) What about trans spliced protein is produced in other tissues, considering that good tropism of AAV8 also for muscle?

We can't address this point since our constructs carry the hybrid liver promoter (HLP) which has been demonstrated to be highly specific for hepatocytes (McIntosh et al, 2013). Otherwise, protein trans splicing (PTS) is a mechanism that can occur in various tissues, like we have shown in the retina (Tornabene et al, 2019),and therefore also in muscle.

ii) In WB of Figure 2-B and 2-C and Figure 3-A and 3-B, second panel, arrows and MW can help to clearly identifying relevant protein bands.

We added arrows and molecular weights in new Figure 3-A and 3-B (old Figure 2-B and 2-C) and in new Figure 4-A and 4-B (old Figure 3-A and 3-B).

Referee #2 (Remarks for Author):

Although currently tested in multiple clinical trials, AAV gene therapy for hemophilia A is complicated by the size of FVIII and the packaging limit of AAV vectors. Esposito et al offer a solution to this problem by engineering of a dual vector expressing FVIII heavy and light chains (of the highly active N6 FVIII variant) fused to intein sequences, so that protein splicing restores the complete molecule. The authors show that correctly spliced B domain-deleted N6-FVIII is produced, which has biological activity. An important finding is that very little antibody is formed against the N6 variant after liver gene transfer in immune competent hemophilia A mice.

While a nice contribution to the field, there are several limitations:

1. Different doses of vector were used for the different variants when compared in vivo, complicating conclusions about efficacy.

The overall objective of this study was to test the therapeutic potential of AAV-intein-F8 in mice. We have optimized F8-N6 (codop F8-N6) to be delivered at low vector doses,and show that indeed it reaches similar therapeutic efficacy than AAV8-V3 given at higher vector doses, therefore reducing both its manufacturing and risk burden. In other words, the in vivo studies were designed to be performed at different doses to show the advantages of the AAV-F8N6 intein product.

It does not appear that the intein approach provides much of an improvement in efficacy.
Codon optimization does not really improved intein-N6 in vivo.

The therapeutic advantage of AAV-F8-N6 intein should be considered relative to the vector dose administered which was lower than that of AAV-F8-V3 but resulted in similar therapeutic efficacy with significantly better coagulation time (by aPTT) and a trend towards shorter bleeding time than in AAV8-F8-V3 animals. Therefore, AAV-F8-N6 reaches similar therapeutic efficacy as AAV-F8-V3 with lower vector doses, lower levels of circulating antibodies and intact vector genomes.

2. Efficiency of proteins trans-splicing is unclear.

We have added to the manuscript a new experiment which allows to infer the efficiency of intein-mediated protein-trans-splicing in liver. To do this we have a used the eGFP reporter protein whose size allows to package it within the same expression cassette both in a single and in two separate AAV-intein vectors. By Western-blot quantification analysis of AAV-treated liver lysates, we show that AAV-intein reconstitute 76% of the full-length eGFP protein expressed from the single AAV vector. These data are now included in the Results section ***AAV-intein mediated protein trans-splicing in mouse liver****, lines 90-104.*

It is unclear if there is a chain imbalance and how much unspliced single chains are generated and secreted.

Quantification following Western-blot analysis of lysates of HEK293 cells transfected with both AAV-intein plasmids. Quantification of the single halves after PTS shows that the 5'(~123 kDa) and 3'(~95 kDa) half are 5- and 4- fold more abundant than the full-length N6, respectively. These data are now included in the Results section ***AAV-intein-mediated protein trans-splicing efficiently reconstitutes N6 in-vitro****, lines 165-167.*

3. The reason for lack of anti-FVIII responses against intein-N6 FVIII is unknown.

4. Cytokine data on serum samples are not informative and should be removed.

In this revised version of the manuscript, we have extended the analysis of serum cytokine levels to include early time points (4 and 8 weeks post-injection) when we also detect high levels of anti-F8 antibodies in the AAV-CodopV3-treated mice. This shows significantly increased levels of the two pro-inflammatory cytokines interferon gamma induced protein 10 (IP-10) and eotaxin in the AAV-CodopV3 but not in the AAV- N6 groups starting at 4 weeks post-injection. On the other hand, potentially anti-inflammatory cytokines such as IL-10 and TGF-ß were not increased in the AAV-N6 intein-treated animals, suggesting that N6 is rather immunologically inert at the vector doses tested when compared to V3. We added this data to the Results section ***Mice injected with AAV-CodopV3 develop anti-F8 antibodies****, lines 243-250.*
Understanding the immunogenicity of V3, which we show has limitations in terms of AAV liver gene therapy when compared to N6, goes beyond the scope of this manuscript. However, we have added a paragraph to the Discussion, lines 280-287 where we speculate about the hypotheses behind the F8-V3 immunogenicity.

Referee #3 (Remarks for Author):

In this manuscript, Esposito and co-workers report on an application of the dual intein system for gene therapy of haemophilia A. The authors take advantage of the natural protein trans-splicing property of trans-inteins to reconstitute the expression and secretion of a large, engineered version of F8 in liver, reaching a therapeutically relevant circulating concentration of this factor. Of interest, this approach did not elicit production of anti-F8 antibodies, which were instead observed in the AAV treatments currently under clinical investigation for haemophilia A.

The manuscript is interesting and most of the results are sound. The demonstration that F8 can be expressed in the liver using AAV vectors to express split portions of F8 through the intein system is interesting in itself for haemophilia A, but can also pave the way to other gene therapy applications with inserts longer than the packaging capability of AAV.

The following is a list of suggestions for improvement.

1) A clear indication of the difference in activity of the wild-type form of F8 compared

to the N6, V3 and SQ variants is missing. The authors should specify what they mean with "activity" and better explain such differences.

2) Page 8 line 144. If the activity assay is so variable, how were the authors able to decide which variant was the best? Is there a more reliable way to define the differences among the three variants?

Our selection of N6 over the other variants was based on the trend towards higher levels of F8 activity (by chromogenic assay) measured in the medium of transfected cells (Fig 2B). We acknowledge that the assay to measure F8 activity is quite variable and we have analyzed the medium of transfected cells with an enzyme-linked immunosorbent assay (ELISA) which shows that N6 is more abundant than the other variants. This new data are now included in the Results section ***In vitro characterization of human F8 variants****, lines 135-140 and Fig Expanded View 2.*

3) Page 5 line 155. The splitting point was chosen within the B-domain. Is this related to the occasionally different spliced products that are generated?

The selection of the splitting point was based on several considerations: i. protein trans-splicing requires the presence of either Cysteine, Serin or Threonine as the first residue in the 3' half of the coding sequence; ii. the two halves are of similar size to fit into AAV; iii. the splitting point was selected within the dispensable B domain to preserve the integrity of other less dispensable F8 domains.

4) Fig 3 A-B. The immunoblotting with the dual vector combination shows a band of the expected size but also other weak but visible bands with different mobility. Are these other spliced products?

They are spliced products as they are present in the lysates of cells transfected with the single halves. Please note that according to the new version of the manuscript Fig 3A-B is now Fig 4A-B.

How could these products interfere with F8 expression?

As full-length F8 is not expressed independently but is the result of protein trans-splicing between the 5' and 3' halves which are independently transcribed and translated from AAV vectors, the two halves are not expected to interfere with F8 protein levels.
We have confirmed this by transfecting the full-length F8N6 plasmid into HEK293 cells (N=3, figure below) together with plasmids either encoding for the 5' or the 3' F8N6. Western blot quantification of cell lysates shows that the levels of full-length F8 were similar among the various experimental conditions.

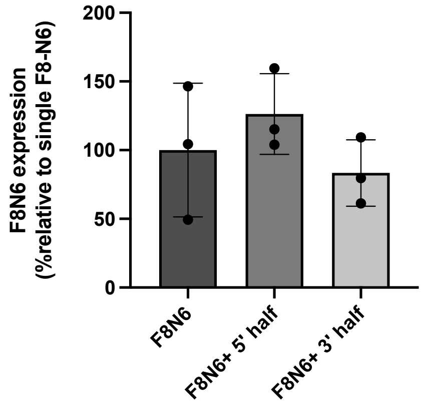

Legend: Western blot quantification shows similar F8N6 protein expression between F8N6 = single plasmid carrying the full-length F8N6; F8N6+5 half'= single plasmid carrying the full-length N6 + 5' half of N6; F8N6+3' half= Single plasmid carrying the full-length N6 + 3' half of N6.

5) The authors report that treatment with F8-N6 did not elicit the production of anti-F8 antibodies. This is an interesting observation, but lacks a molecular explanation, or, at least, an attempt at it. Could vector oversize (F8-V3) be a determinant for antibody response? This point is important, as this difference is pivotal as compared to other treatments.

F8-V3 vector oversize can be considered an explanation for the F8-V3 immunogenicity. While this hypothesis is quite difficult to prove, we have added a comment on this in the Discussion section, lines: 285-287.

6) Can the authors comment on the possible immunogenicity of the half size proteins produced by the dual AAV vectors?

In principle half-size proteins can modulate immune-responses, however, animals treated with AAV-intein vectors who express both full-length and half-size proteins have no detectable increase in either anti-inflammatory/immune-tolerogenic or pro-inflammatory cytokines (see Results section ***Mice injected with AAV-CodopV3 develop anti-F8 antibodies****, lines 243-250) suggesting that full-length F8-N6 or its half size proteins are rather inert when compared to F8-V3 at the AAV vector doses we have investigated.*

7) The immunoblottings should report the relative molecular weights, as these appear to be important in this context.

We added the molecular weights in the immunoblottings.

28th Mar 2022

Dear Prof. Auricchio,

Thank you for the submission of your revised manuscript to EMBO Molecular Medicine. I am pleased to inform you that we will be able to accept your manuscript pending the following final amendments:

1) Figures: Please provide main figures and EV figures as high-resolution TIFF, PDF or EPS files. Please check our Author Guidelines: https://www.embopress.org/page/journal/17574684/authorguide#figureformat
2) In the main manuscript file, please do the following:
- Correct/answer the track changes suggested by our data editors by working from the attached document.
- In M&M, statistical paragraph should reflect all information that you have filled in the Authors Checklist, especially regarding randomization, blinding, replication. Please place all exact p-values in an Appendix supplementary table, label it Appendix Table S1 and refer to it in an appropriate place in the figure legends. Please remove the statistical analysis section with p-values from main manuscript text. Statistical test used should be defined in the figure legend.
- Please be aware that all data from large-scale datasets deposited in one of the relevant databases should be made freely available prior the publication of the manuscript. Use the following format to report the accession number of your data:

The datasets produced in this study are available in the following databases:
[data type]: [full name of the resource] [accession number/identifier] ([doi or URL or identifiers.org/DATABASE:ACCESSION])

Please check "Author Guidelines" for more information.
https://www.embopress.org/page/journal/17574684/authorguide#availabilityofpublishedmaterial
3) Conflict of interest: Rename "Conflict of interest" to "Disclosure Statement & Competing Interests". We updated our journal's competing interests policy in January 2022 and request authors to consider both actual and perceived competing interests. Please disclose your relationship with EMBO in the author disclosure statement using the phrase, "Alberto Auricchio is an editorial advisory board. This has no bearing on the editorial consideration of this article for publication." Please review the policy https://www.embopress.org/competing-interests and update your competing interests if necessary.
4) Synopsis:
- Synopsis image: Please provide a striking image or visual abstract as a high-resolution jpeg file 550 px-wide x (250-400)-px high to illustrate your article.
- Please check your synopsis text and image and submit their final versions with your revised manuscript. Please be aware that in the proof stage minor corrections only are allowed (e.g., typos).
5) For more information: There is space at the end of each article to list relevant web links for further consultation by our readers. Could you identify some relevant ones and provide such information as well? Some examples are patient associations, relevant databases, OMIM/proteins/genes links, author's websites, etc...
6) As part of the EMBO Publications transparent editorial process initiative (see our Editorial at http://embomolmed.embopress.org/content/2/9/329), EMBO Molecular Medicine will publish online a Review Process File (RPF) to accompany accepted manuscripts. This file will be published in conjunction with your paper and will include the anonymous referee reports, your point-by-point response and all pertinent correspondence relating to the manuscript. Let us know whether you agree with the publication of the RPF and as here, if you want to remove or not any figures from it prior to publication. Please note that the Authors checklist will be published at the end of the RPF.
7) Please provide a point-by-point letter INCLUDING my comments as well as the reviewer's reports and your detailed responses (as Word file).

I look forward to reading a new revised version of your manuscript as soon as possible.

Yours sincerely,

Zeljko Durdevic

Zeljko Durdevic
Editor
EMBO Molecular Medicine

***** Reviewer's comments *****

Referee #1 (Comments on Novelty/Model System for Author):

Consistently with my previous review, I confirm the overall positive opinion on this manuscript. The perplexities I previously raised were overcome by the comments of the authors, who better highlighted the value of their work.

Referee #1 (Remarks for Author):

The authors addressed most of my comments with exhaustive discussions. Overall the revision is well organized and clearly presented and now several comments and clarifications are included in the manuscript. The overall quality of the paper is high and represents an interesting contribution to gene therapy for HemA.
In my opinion, in the present form this paper is worth to be published in EMBO Molecular Medicine

In my opinion, in the present form this paper is worth to be published in EMBO Molecular Medicine.

Referee #3 (Remarks for Author):

The authors made a commendable job at responding to the reviewers' comments. I find their arguments quite convincing and the modifications introduced in this revised version of the manuscript appropriate. I have no further comments to add.

The authors performed the requested editorial changes.

2nd Revision - Editorial Decision 11th Apr 2022

We are pleased to inform you that your manuscript is accepted for publication and is now being sent to our publisher to be included in the next available issue of EMBO Molecular Medicine.

EMBO Press Author Checklist

Corresponding Author Name: Alberto Auricchio	
Journal Submitted to: EMBO Molecular Medicine	
Manuscript Number: 15199	

USEFUL LINKS FOR COMPLETING THIS FORM
The EMBO Journal - Author Guidelines
EMBO Reports - Author Guidelines
Molecular Systems Biology - Author Guidelines
EMBO Molecular Medicine - Author Guidelines

Reporting Checklist for Life Science Articles (updated January 2022)
This checklist is adapted from Materials Design Analysis Reporting (MDAR) Checklist for Authors. MDAR establishes a minimum set of requirements in transparent reporting in the life sciences (see Statement of Task: 10.31222/osf.io/9sm4x). Please follow the journal's guidelines in preparing your manuscript.
Please note that a copy of this checklist will be published alongside your article.

Abridged guidelines for figures
1. Data
The data shown in figures should satisfy the following conditions:
- the data were obtained and processed according to the field's best practice and are presented to reflect the results of the experiments in an accurate and unbiased manner.
- ideally, figure panels should include only measurements that are directly comparable to each other and obtained with the same assay.
- plots include clearly labeled error bars for independent experiments and sample sizes. Unless justified, error bars should not be shown for technical replicates.
- if n<5, the individual data points from each experiment should be plotted. Any statistical test employed should be justified.
- Source Data should be included to report the data underlying figures according to the guidelines set out in the authorship guidelines on Data Presentation.

2. Captions
Each figure caption should contain the following information, for each panel where they are relevant:
- a specification of the experimental system investigated (eg cell line, species name).
- the assay(s) and method(s) used to carry out the reported observations and measurements.
- an explicit mention of the biological and chemical entity(ies) that are being measured.
- an explicit mention of the biological and chemical entity(ies) that are altered/varied/perturbed in a controlled manner.
- the exact sample size (n) for each experimental group/condition, given as a number, not a range;
- a description of the sample collection allowing the reader to understand whether the samples represent technical or biological replicates (including how many animals, litters, cultures, etc.).
- a statement of how many times the experiment shown was independently replicated in the laboratory.
- definitions of statistical methods and measures:
  - common tests, such as t-test (please specify whether paired vs. unpaired), simple χ2 tests, Wilcoxon and Mann-Whitney tests, can be unambiguously identified by name only, but more complex techniques should be described in the methods section;
  - are tests one-sided or two-sided?
  - are there adjustments for multiple comparisons?
  - exact statistical test results, e.g., P values = x but not P values < x;
  - definition of 'center values' as median or average;
  - definition of error bars as s.d. or s.e.m.

Please complete ALL of the questions below.
Select "Not Applicable" only when the requested information is not relevant for your study.

Materials

Newly Created Materials	Information included in the manuscript?	In which section is the information available? (Reagents and Tools Table, Materials and Methods, Figures, Data Availability Section)
New materials and reagents need to be available; do any restrictions apply?	Not Applicable	

Antibodies	Information included in the manuscript?	In which section is the information available? (Reagents and Tools Table, Materials and Methods, Figures, Data Availability Section)
For **antibodies** provide the following information:<br>- Commercial antibodies: RRID (if possible) or supplier name, catalogue number and or/clone number<br>- Non-commercial: RRID or citation	Yes	Materials and Methods, Western blot analysis paraghap: page 19.

DNA and RNA sequences	Information included in the manuscript?	In which section is the information available? (Reagents and Tools Table, Materials and Methods, Figures, Data Availability Section)
**Short novel DNA or RNA including primers, probes**: provide the sequences.	Not Applicable	

Cell materials	Information included in the manuscript?	In which section is the information available? (Reagents and Tools Table, Materials and Methods, Figures, Data Availability Section)
**Cell lines:** Provide species information, strain. Provide accession number in repository **OR** supplier name, catalog number, clone number, and/**OR** RRID.	Not Applicable	
**Primary cultures:** Provide species, strain, sex of origin, genetic modification status.	Not Applicable	
Report if the cell lines were recently **authenticated** (e.g., by STR profiling) and tested for mycoplasma contamination.	Not Applicable	

Experimental animals	Information included in the manuscript?	In which section is the information available? (Reagents and Tools Table, Materials and Methods, Figures, Data Availability Section)
**Laboratory animals or Model organisms:** Provide species, strain, sex, age, genetic modification status. Provide accession number in repository **OR** supplier name, catalog number, clone number, **OR** RRID.	Yes	Materials and Methods. Animal model paragraph: page 20. Retro-orbital injection of AAV vectors in mice paragraph: page 21.
**Animal observed in or captured from the field:** Provide species, sex, and age where possible.	Yes	Materials and Methods. Animal model paragraph: page 20. Retro-orbital injection of AAV vectors in mice paragraph: page 21.
Please detail **housing and husbandry conditions**.	Yes	Materials and Methods. Animal model paragraph: page 20.

Plants and microbes	Information included in the manuscript?	In which section is the information available? (Reagents and Tools Table, Materials and Methods, Figures, Data Availability Section)
**Plants:** provide species and strain, ecotype and cultivar where relevant, unique accession number if available, and source (including location for collected wild specimens).	Not Applicable	
**Microbes:** provide species and strain, unique accession number if available, and source.	Not Applicable	

Human research participants	Information included in the manuscript?	In which section is the information available? (Reagents and Tools Table, Materials and Methods, Figures, Data Availability Section)
If collected and within the bounds of privacy constraints report on age, sex and gender or ethnicity for all study participants.	Not Applicable	

Core facilities	Information included in the manuscript?	In which section is the information available? (Reagents and Tools Table, Materials and Methods, Figures, Data Availability Section)
If your work benefited from core facilities, was their service mentioned in the acknowledgments section?	Yes	Ackonwledgments section: page 26.

Design

Study protocol	Information included in the manuscript?	In which section is the information available? (Reagents and Tools Table, Materials and Methods, Figures, Data Availability Section)
If study protocol has been **pre-registered, provide DOI in the manuscript**. For clinical trials, provide the trial registration number **OR** cite DOI.	Not Applicable	
Report the **clinical trial registration number** (at ClinicalTrials.gov or equivalent), where applicable.	Not Applicable	

Laboratory protocol	Information included in the manuscript?	In which section is the information available? (Reagents and Tools Table, Materials and Methods, Figures, Data Availability Section)
Provide DOI OR other citation details if **external detailed step-by-step protocols** are available.	Yes	Materials and Methods. AAV vector production and characterization paragraph: page 18.

Experimental study design and statistics	Information included in the manuscript?	In which section is the information available? (Reagents and Tools Table, Materials and Methods, Figures, Data Availability Section)
Include a statement about **sample size** estimate even if no statistical methods were used.	Yes	Materials and Methods. Statistical analysis paragraph: pages 16-17.
Were any steps taken to minimize the effects of subjective bias when allocating animals/samples to treatment (e.g. **randomization procedure**)? If yes, have they been described?	Yes	Materials and Methods. Statistical analysis paragraph: page 16.
Include a statement about **blinding** even if no blinding was done.	Yes	Materials and Methods. Statistical analysis paragraph: page 16.
Describe **inclusion/exclusion criteria** if samples or animals were excluded from the analysis. Were the criteria pre-established?<br><br>If sample or data points were omitted from analysis, report if this was due to attrition or intentional exclusion and provide justification.	Yes	Materials and Methods.Study design paragraph and statistical analysis paragraph: pages 16-17.
For every figure, are **statistical tests** justified as appropriate? Do the data meet the assumptions of the tests (e.g., normal distribution)? Describe any methods used to assess it. Is there an estimate of variation within each group of data? Is the variance similar between the groups that are being statistically compared?	Yes	Materials and Methods. Statistical analysis paragraph: pages 16-17.

Sample definition and in-laboratory replication	Information included in the manuscript?	In which section is the information available? (Reagents and Tools Table, Materials and Methods, Figures, Data Availability Section)
In the figure legends: state number of times the experiment was **replicated** in laboratory.	Yes	Figure legends section: pages 33-39.
In the figure legends: define whether data describe **technical or biological replicates**.	Yes	Figure legends section: pages 33-39.

Ethics

Ethics	Information included in the manuscript?	In which section is the information available? (Reagents and Tools Table, Materials and Methods, Figures, Data Availability Section)
Studies involving **human participants**: State details of **authority granting ethics approval** (IRB or equivalent committee(s), provide reference number for approval.	Not Applicable	
Studies involving **human participants**: Include a statement confirming that **informed consent** was obtained from all subjects and that the experiments conformed to the principles set out in the WMA Declaration of Helsinki and the Department of Health and Human Services Belmont Report.	Not Applicable	
Studies involving **human participants:** For publication of **patient photos**, include a statement confirming that consent to publish was obtained.	Not Applicable	
Studies involving experimental **animals**: State details of **authority granting ethics approval** (IRB or equivalent committee(s), provide reference number for approval. Include a statement of compliance with ethical regulations.	Yes	Materials and Methods. Retro-orbital injection of AAV vectors in mice paraghaph: page 20.
Studies involving **specimen and field samples:** State if relevant **permits** obtained, provide details of authority approving study; if none were required, explain why.	Not Applicable	

Dual Use Research of Concern (DURC)	Information included in the manuscript?	In which section is the information available? (Reagents and Tools Table, Materials and Methods, Figures, Data Availability Section)
Could your study fall under dual use research restrictions? Please check biosecurity documents and list of **select agents and toxins** (CDC): https://www.selectagents.gov/sat/list.htm	Not Applicable	
If you used a select agent, is the security level of the lab appropriate and reported in the manuscript?	Not Applicable	
If a study is subject to dual use research of concern regulations, is the name of the **authority granting approval and reference number** for the regulatory approval provided in the manuscript?	Not Applicable	

Reporting

The MDAR framework recommends adoption of discipline-specific guidelines, established and endorsed through community initiatives. Journals have their own policy about requiring specific guidelines and recommendations to complement MDAR.

Adherence to community standards	Information included in the manuscript?	In which section is the information available? (Reagents and Tools Table, Materials and Methods, Figures, Data Availability Section)
State if relevant guidelines or checklists (e.g., **ICMJE, MIBBI, ARRIVE, PRISMA**) have been followed or provided.	Not Applicable	
For **tumor marker prognostic studies**, we recommend that you follow the **REMARK** reporting guidelines (see link list at top right). See author guidelines, under 'Reporting Guidelines'. Please confirm you have followed these guidelines.	Not Applicable	
For **phase II and III randomized controlled trials**, please refer to the **CONSORT** flow diagram (see link list at top right) and submit the CONSORT checklist (see link list at top right) with your submission. See author guidelines, under 'Reporting Guidelines'. Please confirm you have submitted this list.	Not Applicable	

Data Availability

Data availability	Information included in the manuscript?	In which section is the information available? (Reagents and Tools Table, Materials and Methods, Figures, Data Availability Section)
Have **primary datasets** been deposited according to the journal's guidelines (see 'Data Deposition' section) and the respective accession numbers provided in the Data Availability Section?	Yes	Data Availability Section: page 27.
Were **human clinical and genomic datasets** deposited in a public access-controlled repository in accordance to ethical obligations to the patients and to the applicable consent agreement?	Not Applicable	
Are **computational models** that are central and integral to a study available without restrictions in a machine-readable form? Were the relevant accession numbers or links provided?	Not Applicable	
If publicly available data were reused, provide the respective **data citations in the reference list**.	Not Applicable	
